# VirB, a key transcriptional regulator of *Shigella* virulence, requires a CTP ligand for its regulatory activities

**DOI:** 10.1128/mbio.01519-23

**Published:** 2023-09-20

**Authors:** Taylor M. Gerson, Audrey M. Ott, Monika M. A. Karney, Jillian N. Socea, Daren R. Ginete, Lakshminarayan M. Iyer, L. Aravind, Ronald K. Gary, Helen J. Wing

**Affiliations:** 1 School of Life Sciences, University of Nevada Las Vegas, Las Vegas, Nevada, USA; 2 Computational Biology Branch, National Library of Medicine, Bethesda, Maryland, USA; 3 Department of Chemistry and Biochemistry, University of Nevada Las Vegas, Las Vegas, Nevada, USA; Pennsylvania State University, University Park, Pennsylvania, USA

**Keywords:** bacterial gene regulation, large plasmids, transcriptional silencing/anti-silencing, ParB/Spo0J, GFP fusions, focus formation, Congo red phenotype, plasmid partitioning

## Abstract

The VirB protein, encoded by the large virulence plasmid of *Shigella* spp., is a key transcriptional regulator of virulence genes. Without *virB*, *Shigella* cells are avirulent. On the virulence plasmid, VirB functions to offset transcriptional silencing mediated by the nucleoid structuring protein, H-NS, which binds and sequesters AT-rich DNA, making it inaccessible for gene expression. Thus, gaining a mechanistic understanding of how VirB counters H-NS-mediated silencing is of considerable interest. VirB is unusual in that it does not resemble classic transcription factors. Instead, its closest relatives are found in the ParB superfamily, where the best-characterized members function in faithful DNA segregation before cell division. Here, we show that VirB is a fast-evolving member of this superfamily and report for the first time that the VirB protein binds a highly unusual ligand, CTP. VirB binds this nucleoside triphosphate preferentially and with specificity. Based on alignments with members of the ParB family, we identify amino acids of VirB likely to bind CTP. Substitutions in these residues disrupt several well-documented activities of VirB, including its anti-silencing activity, its ability to generate a Congo red positive phenotype, and the ability of the VirB protein to form foci in the bacterial cytoplasm when fused to GFP. Although stably produced, our CTP binding mutants do not bind DNA *in vivo*, likely explaining these phenotypes. Thus, this work is the first to show that VirB is a bona fide CTP-binding protein, links *Shigella* virulence phenotypes to the nucleoside triphosphate CTP, and provides novel insight into VirB-CTP-DNA interactions.

**IMPORTANCE:**

*Shigella* species cause bacillary dysentery, the second leading cause of diarrheal deaths worldwide. There is a pressing need to identify novel molecular drug targets. *Shigella* virulence phenotypes are controlled by the transcriptional regulator, VirB. We show that VirB belongs to a fast-evolving, plasmid-borne clade of the ParB superfamily, which has diverged from versions with a distinct cellular role—DNA partitioning. We report that, like classic members of the ParB family, VirB binds a highly unusual ligand, CTP. Mutants predicted to be defective in CTP binding are compromised in a variety of virulence attributes controlled by VirB, likely because these mutants cannot engage DNA. This study (i) reveals that VirB binds CTP, (ii) provides a link between VirB-CTP interactions and *Shigella* virulence phenotypes, (iii) provides new insight into VirB-CTP-DNA interactions, and (iv) broadens our understanding of the ParB superfamily, a group of bacterial proteins that play critical roles in many bacteria.

## INTRODUCTION

*Shigella* spp*.* are intracellular bacterial pathogens and the causative agents of bacillary dysentery (shigellosis) (1 – 4). Upon entry into the human host and in response to body temperature, a transcriptional cascade (5), leads to the production of VirB (also called InvE). VirB is a key transcriptional regulator of genes found on the large virulence plasmid (pINV) (6, 7) which directly or indirectly leads to the upregulation of about 50 genes (7 – 9), including those encoding the type III secretion system, other crucial virulence-associated factors (OspZ, OspD1, and IcsP (10 – 12), and the transcriptional activator, MxiE, and its co-activator, IpgC (13, 14). Consequently, VirB-dependent gene regulation is essential for *Shigella* virulence (7). In the late 1970s/early 1980s, *Shigella* virulence was found to directly correlate with the ability of colonies to bind the organic dye, Congo red (15 – 17). Mutants lacking *virB*, exhibit a Congo red negative (CR-) phenotype (18, 19), providing an easy way to evaluate the activity of VirB and assess *Shigella* virulence.

VirB does not function like a traditional transcription factor, rather, it functions as an anti-silencing protein, alleviating transcriptional silencing mediated by the histone-like nucleoid structuring protein, H-NS (10, 12, 20 – 25). H-NS coats and condenses DNA by binding to the minor groove of AT-rich DNA, which is a common feature of horizontally acquired DNA (26, 27). Thus, H-NS serves as a xenogeneic silencer of newly acquired DNA (28 – 32), which includes many important virulence genes in a variety of bacterial pathogens (33 – 37). Alleviation of H-NS-mediated silencing is caused by VirB engaging its DNA recognition site and spreading along DNA (24), which is thought to remodel H-NS-DNA complexes, allowing previously inaccessible or transcriptionally non-permissive DNA to be bound by RNA polymerase (25). Recent work suggests that anti-silencing or the remodeling of H-NS-DNA complexes is likely mediated by a localized loss of negative DNA supercoils, which is triggered when VirB engages its site and spreads along DNA, causing a stiffening of the helix (38).

VirB is not related to classic bacterial transcriptional regulators but is a member of the ParB superfamily (20, 39 – 41). Most members of the ParB superfamily are characterized to play key roles in chromosome or plasmid segregation prior to cell division (42 – 45). While VirB does not function in DNA segregation (41, 46, 47), we hypothesize that its evolutionary relationship to ParB proteins will provide insight into its mechanism of anti-silencing. ParB proteins load onto DNA at palindromic DNA recognition sites, called *parS* (48 – 50), and condense adjacent DNA regions into large nucleoprotein complexes (51). Evidence suggests that these nucleoprotein complexes are formed by the lateral spreading of ParB proteins along DNA into regions flanked by *parS* (52 – 55). After ParB proteins establish partitioning complexes, an ATPase, ParA, is recruited to the complex and interacts with the N-terminus of ParB to move sister replicons to the poles of the cell through a ratchet-like mechanism (56 – 58). Of note, functional GFP-ParB fusions form fluorescent foci at the pole prior to cell division. Recently, some members of the ParB superfamily have been shown to bind and hydrolyze CTP. The CTP ligand is needed for an open-to-closed conformational change, which allows ParB to dissociate from the *parS* site and spread along DNA (45, 59 – 61).

Like ParB proteins, VirB recognizes a palindromic *parS*-like DNA binding site (20, 22, 24, 41, 62), and multimers of VirB form on DNA both *in vitro* and *in vivo* (39, 40, 63). Both activities are essential for the transcriptional anti-silencing of virulence genes by VirB (24, 39 – 41, 62, 63). However, there are clear differences between VirB and ParB proteins too. Aside from different cellular functions (12, 22, 64), the *virB* locus lacks a *parA*-like gene (43) and the VirB protein does not require a ParA-like partner protein in order to function (20, 46, 65). The recent discovery that some ParB members function as CTP-binding proteins raises questions as to whether VirB binds CTP or some other NTP and what effect this may have on transcriptional anti-silencing. Thus, the overarching goals of this study were to (i) gain further insight into the ParB superfamily and the relationship between classic ParB proteins and VirB, (ii) determine if VirB is capable of binding CTP, and if so, (iii) characterize how CTP binding may impact the virulence phenotypes controlled by VirB.

## RESULTS

### VirB is a member of a fast-evolving clade within the ParB superfamily

**Fig 1:**
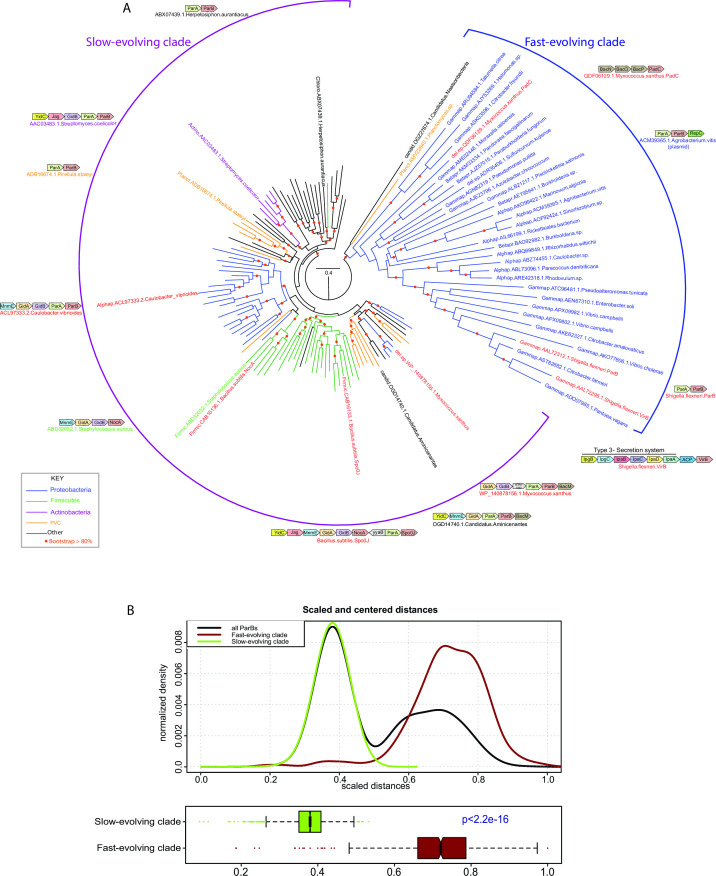
Maximum likelihood tree of ParB protein sequences including the VirB-like fast-evolving sequences. (**A**) Maximum likelihood tree of ParB protein sequences including the VirB-like fast-evolving sequences. The tree was computed using IQtree and bootstrapped with 1,000 replicates. Nodes and branches corresponding to widely represented phylogenetic clades are colored according to the key. Protein sequences are denoted with their clade, gene, and species name. The slow- and fast-evolving clades are marked and representative gene neighborhoods of distinct clades of the tree are shown. Genes in gene neighborhoods are shown as box arrows with the arrowhead pointing to the gene at the 3′ end. Clade abbreviations include: Actino,: Actinomycetes; Alphap, Alpha-proteobacteria; Betapr, Beta-proteobacteria; candid, Dark-matter bacteria; Chloro, Chloroflexi; del.ep, Delta/epsilon proteobacteria; Firmic, Firmicutes; Gammap, Gamma-proteobacteria; Planct, Planctomycetes. (**B**) Differential evolutionary rates derived from pairwise centered and scaled maximum-likelihood distances shown as a kernel density and box plots.

To understand the provenance and potential biochemical functions of *Shigella* VirB, we recovered both immediate and more distant homologs (see Materials and Methods). Searches typically retrieved several VirB-related ParB superfamily members, e.g., *Myxococcus xanthus* PadC and *Agrobacterium vitis* ParB from the pAtS4c plasmid as top hits, and then recovered the classic chromosome partitioning ParB proteins. A representative sequence set was used to construct a multiple alignment to analyze the sequence conservation patterns and for phylogenetic analysis. The phylogenetic trees revealed a consistent and well-supported topology in which *Shigella* VirB and a group of predominantly proteobacterial proteins formed a distinct clade to the exclusion of the remaining classic bacterial ParB proteins (Fig. 1A; Fig. S1). This separation was corroborated by a clear bimodal distribution of the scaled and centered distances of the classic ParB from cellular chromosomes and VirB-related proteins. The former constituted a peak at the left of the distribution with short distances, and the latter right peak corresponded to large distances (Fig. 1B). The mean scaled and centered distances are significantly higher for the VirB-like clade as opposed to the classic cellular-chromosome-encoded ParBs (*P* < 2.2 × 10^−16^), indicating that the VirB-like clade is fast-evolving relative to the classic ParBs (the slow-evolving clade).

These cladal differences are also reflected in gene-neighborhood contexts (Fig. 1A; Fig. S1). Across bacteria, classic ParBs from cellular chromosomes tend to be encoded in largely conserved gene neighborhoods along with genes encoding: (i) GidA and MnmE, both of which are involved in the synthesis of the carboxymethylaminomethyl modification of uridine 34 of tRNAs (66, 67); (ii) GidB, the 16S rRNA guanidine 535 N7 methylase (68, 69); (iii) YidC, the membrane protein assembly factor (70, 71); (iv) Jag, an RNA-binding protein with KH and R3H domains that regulates the expression of the cell-division protein FtsA post-transcriptionally (72); and (v) ParA, the ATPase partner of ParB required for chromosome segregation (50, 57, 73, 74). Less frequently, these neighborhoods might also code for one or more genes for the β-helix cytoskeletal protein bactofilin, implicated in cell division along with ParA and ParB (75 – 78). In sharp contrast, other than the neighborhood association with genes encoding ParA and, on rare occasions, bactofilin (e.g., *M. xanthus* PadC), the fast-evolving VirB-like clade lacks the remaining associations of classic ParBs. Additionally, the fast-evolving clade is primarily found on free plasmids or plasmids integrated into cellular chromosomes. These findings suggest that, with the exception of partnering with ParA, the fast-evolving VirB-like ParBs do not have the genetic and physical interaction constraints that act on the classic ParBs, which has allowed them to evolve more rapidly.

Both the classic ParBs and the VirB-like clade possess a conserved domain architecture with four evolutionarily distinct domains, including (i) a ParB NTP-binding/hydrolyzing domain; (ii) a DNA-binding helix-turn-helix domain; (iii) a tetra-helical bundle comprised of two α-α-hairpins; and (iv) a C-terminal domain likely playing a role both in dimerization and DNA binding. Together they form a dimer with a central aperture through which DNA can be threaded. Additionally, they contain an unstructured N-terminal tail with two conserved arginines that are proposed to act as “arginine fingers” to facilitate ATP-hydrolysis by the ParA partner protein. This N-terminal tail is a diagnostic feature of ParB superfamily proteins that interacts with a ParA partner (Fig. S2). Notably, we found that this tail has diverged considerably and lost the arginines in VirB, consistent with the lack of a ParA partner interaction (Fig. S2). However, VirB is nested in the tree with versions that do interact with a ParA protein (e.g., ParB from *Shigella flexneri* plasmid pCP301). This suggests a two-step model for the emergence of VirB from a ParB-like precursor. First, release from the constraints typical of the chromosomal classic ParBs allowed the plasmid-borne versions to rapidly evolve and diverge. This divergence allowed the exploration of new functions, such as transcriptional regulation, which utilized the ancestral DNA-binding properties of ParB for a different role. This favored the loss of the determinants of its original function, i.e., the ParA-interaction residues. Indeed, multimer modeling via the AlphaFold2 program indicated that while the slow-evolving ParBs form a predicted complex with ParA via the N-terminal tail, *Shigella* VirB did not yield any such complex.

### The *Shigella* anti-silencing protein VirB binds an unusual biological ligand, CTP

**Fig 2:**
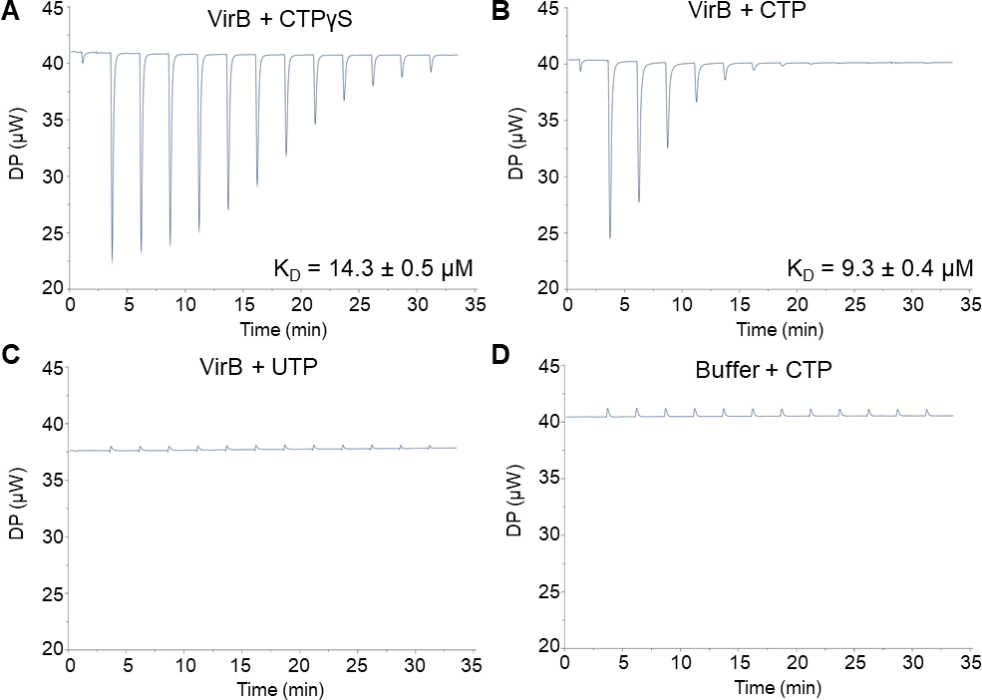
The *Shigella* anti-silencing protein VirB binds a novel ligand, CTP. ITC measurements with (**A**) 90 µM VirB-His6 and 3 mM CTPγS, (**B**) 45 µM VirB-His6 and 3 mM CTP, (**C**) 45 µM VirB-His6 and 3 mM UTP, and (**D**) VirB binding buffer and 3 mM CTP. In panels B and C, the VirB protein concentration was lowered to 45 µM to reach saturation and conserve protein.

Despite its overall rapid divergence, all members of the fast-evolving clade, including VirB, retain key NTP-binding determinants conserved across the ParB superfamily (Fig. S2) and at least one member of this clade, *M. xanthus* PadC, binds CTP like the classic ParBs (59 – 61). Hence, we next investigated if VirB binds CTP. Using isothermal titration calorimetry (ITC), CTPγS, CTP, or UTP was titrated into a cell containing purified VirB protein, and differential power (DP) was plotted against time (Fig. 2).

During initial experiments, non-hydrolyzable CTP, CTPγS, was titrated into the VirB-containing cell to eliminate possible interference from any potential nucleotide hydrolysis. The addition of CTPγS produced exothermic binding events (downward-facing peaks) that gradually diminished as binding sites became saturated (Fig. 2A). The data were fit to a “one set of sites” (i.e., non-cooperative) binding model, and VirB was calculated to bind CTPγS with a K_D_ value of 14.3 µM ± 0.5 µM, a value similar to those calculated for Spo0J (*Bacillus subtilis*), a classic ParB member (10.3 ± 1.2 µM) (60). Because VirB was not fully saturated by CTPγS after 13 injections, we decreased the VirB concentration by twofold in subsequent experiments. Commercially available CTP was found to bind VirB with affinities similar to those seen with CTPγS (K_D_ = 9.3 µM ± 0.4 µM; Fig. 2B). To determine if VirB binds other pyrimidine triphosphates available in the cell, UTP was used. In contrast to CTP, UTP showed no binding signatures (Fig. 2C), resembling the buffer-only controls (Fig. 2D; Fig. S3A and B). Failure of VirB to bind UTP strongly supports the conclusion that VirB binds CTP with specificity. After the binding sites on VirB were saturated, subsequent injections of CTP produced no thermal signal (Fig. 2B), suggesting VirB is incapable of hydrolyzing CTP under these experimental conditions. Thus, VirB, the anti-silencing protein of *S. flexneri* virulence genes*,* is a bona fide CTP-binding protein and binds CTP with affinities similar to those exhibited by classic ParBs (59 – 61).

### VirB preferentially binds CTP over other NTP ligands found in the cell

Our ITC experiments revealed that VirB binds CTP, but not UTP (Fig. 2). However, to conclusively test whether VirB binds any other nucleoside triphosphates available in the cell, we exploited the differential radial capillary action of ligand assay (DRaCALA) with cold competitor NTPs (79). Briefly, DRaCALA is based on the premise that a small, radiolabeled ligand will diffuse on nitrocellulose, but if bound to a protein of interest, the protein:ligand complex will not diffuse and remain at the original point of contact, resulting in a tight spot of radioactive signal (dark inner core). In our study, we incubated purified VirB protein with radiolabeled (hot) α^32^P-CTP and, where appropriate, excess unlabeled (cold) NTP ligands (CTP, UTP, ATP, or GTP). Reaction mixtures were then spotted onto dry nitrocellulose membranes and air-dried, prior to imaging.

**Fig 3:**
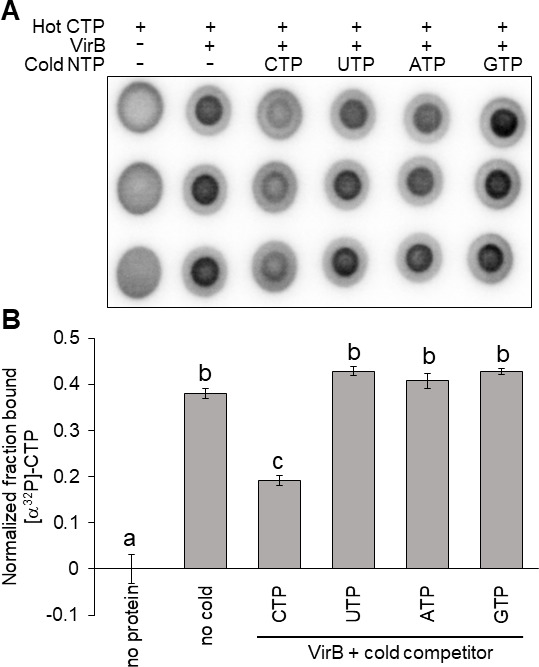
VirB preferentially binds CTP over other NTP ligands found in the cell. (**A**) DRaCALA images of competition assays assessing the ability of 500 µM indicated cold NTP to compete with binding interactions between 5 nM [α^32^P]-CTP and 50 µM VirB-His_6_. Ligand binding is indicated by a darker inner core staining due to rapid immobilization of the VirB-CTP complex. Three independent trials of this experiment were run. Each trial contained three technical replicates. A representative trial is shown (each row represents a technical replicate). (**B**) Graph of normalized fraction bound (F_B_) calculated for each sample in Fig. 3A. Significance was calculated using a one-way ANOVA with *post hoc* Bonferroni, *P* < 0.05. Lowercase letters indicate statistical groups. Complete statistical analysis provided in Table S3.

As expected, in the absence of VirB, but in the presence of radiolabeled (hot) CTP, no dark inner core was detected, yet in the presence of both, a dark inner core was seen. These data indicate that VirB binds the radiolabeled CTP (Fig. 3A), supporting our ITC data. Next, cold competitor NTPs were included in the reaction mix at a 100,000-fold molar excess. When excess cold CTP was added to the reaction mix, the radioactive signal was much more diffuse, indicating that the cold competitor CTP had competed for VirB binding (Fig. 3A). In support of this, a significant decrease in the fraction of radiolabeled CTP bound was quantified, relative to that found in the absence of the competitor (Fig. 3B). While some residual binding of hot CTP to VirB was observed, this was likely caused by the simultaneous addition of the labeled and unlabeled CTP and the high concentration of VirB used in these assays. Importantly and in contrast, when excess cold UTP, ATP, or GTP was added to the reaction mixture, no competition for the VirB protein was observed (Fig. 3A and B). Thus, these results conclusively demonstrate that VirB binds CTP preferentially and with specificity.

### Identification of key VirB residues predicted to be required for CTP binding

**Fig 4:**
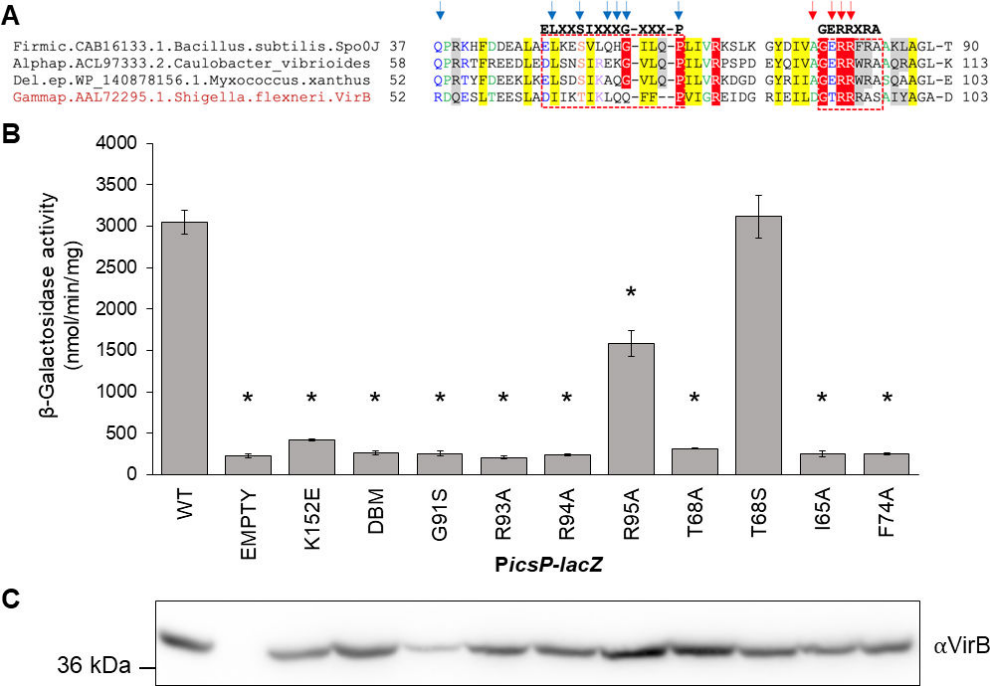
Key residues in the predicted CTP binding pocket are required for the anti-silencing activity of VirB. (**A**) Multiple sequence alignment (MSA of classic ParBs that bind CTP and VirB, generated using the Mafft program with a local pair algorithm. Full alignment and additional details provided in Fig. S2. Taxonomic clade name, Genbank accession, and species name separated by dots are denoted. Consensus abbreviations and coloring scheme are as follows: hydrophobic (FWYILVACM), aromatic (FWY) and aliphatic (ILV) residues shaded yellow; charged (DEHKR) and basic (KRH) residues colored magenta, big (LIFMWYERKQ) residues shaded gray, alcohol-group (ST) residues colored red, polar (STECDRKHNQ) residues colored blue, small (AGSCDNPTV) residues colored green, and tiny (GAS) residues shaded green. Fully conserved residues are shaded red. Arrows indicate nucleotide-binding residues (blue) and catalytic residues (red). (**B**) β-Galactosidase assay used to assess the regulatory activity of pBAD-*virB* derivatives at the VirB-dependent *icsP* promoter. Significance was calculated using a one-way ANOVA with *post hoc* Tukey HSD, *P* < 0.05. *, statistically significant compared to wild type. Complete statistical analysis is provided in Table S4. (**C**) Western Blot analysis using an anti-VirB antibody to assess protein production of pBAD-*virB* mutants alongside a SeeBlue Plus2 Prestained Standard. Assays were completed with three biological replicates and repeated three times. Representative data are shown.

With the discovery that VirB binds CTP, we next wanted to identify amino acid residues in VirB predicted to be required for CTP binding. A multiple sequence alignment (MSA) that included VirB and the well-characterized ParB proteins demonstrated to bind CTP (*B. subtilis* Spo0J [60], *Caulobacter vibriodes* ParB [61], and *M. xanthus* PadC [59]) allowed us to compare residues previously implicated in CTP binding (Fig. 4A; Fig. S2). These are located in two regions (previously described as Box I and Box II [59]) near the N-terminus of their respective proteins (59 – 61). Residues in the motif ELXXSIXXXGXXXP (Box I) were characterized to interact with the cytosine base of CTP, and thus, are critical for CTP binding specificity (59, 80). Whereas, residues in the motif GERRXRA (Box II) were characterized to interact with triphosphate motifs of CTP, playing a role in stabilizing this interaction (59, 80). Our MSA (Fig. 4A; Fig. S2) showed that in Box I, two residues (I65 and F74) correspond to similar residues required for CTP binding in the classic ParBs (Fig. 4A). Additionally, T68, located in Box I, corresponds to a serine in the slow-evolving ParBs, which hydrogen bonds to the cytosine base (Fig. 4A), suggesting T68 might play a comparable role in VirB. In Box II, three residues (G91, R93, and R94) were identified that were fully conserved, when compared to classic ParBs (Fig. 4A). Thus, six residues of interest I65, T68, F74, G91, R93, and R94 were identified. These were explored further in this study.

### Key residues in the predicted CTP binding pocket are required for the anti-silencing activity of VirB

Key VirB residues predicted to bind CTP were targeted for substitution. A suite of *virB* mutant alleles was generated and introduced into the inducible pBAD expression plasmid to generate the following VirB mutants; I65A, T68A, F74A, G91S, R93A, and R94A. Additionally, VirB T68S and VirB R95A were created because we reasoned that T68S would retain the potential for hydrogen bonding at this position, whereas R95A substitutes a non-conserved residue that lies adjacent to a patch of fully conserved core residues.

To assess the impact of these substitutions on VirB-dependent gene regulation, we measured the ability of these VirB mutants to regulate a well-characterized VirB-regulated promoter, P*icsP*. Inducible plasmids each carrying a pBAD-*virB* mutant were introduced into a *virB* mutant derivative of *Shigella flexneri* (AWY3) carrying the P*icsP-lacZ* transcriptional reporter (10, 24), and β-galactosidase activity was measured in cultures following induction. As expected, wild-type VirB had high levels of β-galactosidase activity, indicating that VirB was able to regulate P*icsP*, whereas the empty plasmid control and two well-characterized DNA binding mutants (a single and double mutant [12, 22]) each exhibited low levels of β-galactosidase activity (Fig. 4B). Strikingly, the following VirB mutants I65A, T68A, F74A, G91S, R93A, and R94A exhibited a significant decrease in β-galactosidase activity when compared to wild type and displayed activities similar to the negative controls (Fig. 4B). However, VirB T68S showed no significant decrease (Fig. 4B). The loss of activity by VirB T68A, but the retention by VirB T68S, suggests that the anti-silencing activities of VirB require residue 68, which is predicted to bind CTP, to participate in hydrogen bond formation. Interestingly, VirB R95A showed significantly lower levels of β-galactosidase activity when compared to wild-type VirB, although this activity was significantly higher than that displayed by most of the VirB mutants (Table S4). Thus, even though R95 is not a residue that is conserved in other family members, VirB R95A displayed intermediate levels of anti-silencing activity in our assays (Fig. 4B).

Importantly, western analyses of cell pellets generated from cultures grown identically to those used in our β-galactosidase assays, revealed that all proteins, with one exception, VirB G91S, were made at wild-type levels or higher under assay conditions (Fig. 4C). Since the instability of VirB G91S raised concerns about whether this derivative was structurally sound, it was eliminated from subsequent studies. Taken together, our P*icsP* promoter activity assays and western analyses show that key residues located in the predicted CTP binding pocket of VirB, are required for its transcriptional anti-silencing activity (Fig. 4B and C).

### Key residues in the predicted CTP binding pocket are required for *Shigella* virulence phenotypes

To further examine the effect that our VirB mutants have on virulence phenotypes, we next assessed the Congo red binding of *Shigella* cells expressing the *virB* mutant derivatives. The ability of *Shigella* colonies to bind Congo red is positively correlated with their virulence properties (15, 16). Congo red binding assays were performed using a *virB* mutant derivative of *Shigella flexneri* (AWY3) expressing each of the inducible *virB* mutant alleles from pBAD-*virB* derivatives, as described previously.

**Fig 5:**
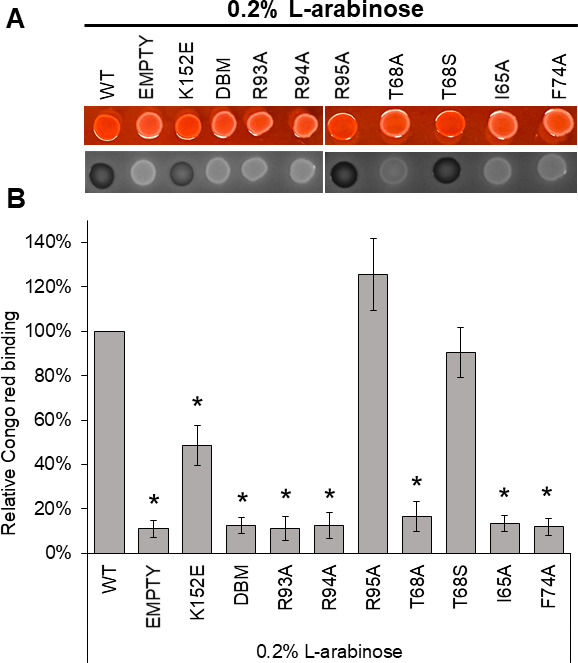
Key residues in the predicted CTP binding pocket are required for *Shigella* virulence phenotype. (**A**) Congo red binding by *S. flexneri virB*::Tn5 harboring pBAD-*virB* derivatives under inducing conditions. Images were captured using visible light (top) and blue light (Cy2) (bottom). (**B**) Quantitative analysis of Congo red binding *S. flexneri virB*::Tn5 harboring pBAD-*virB* derivatives (induced). Relative Congo red binding was calculated as [(OD_498_/OD_600_)/(average (OD_498_/OD_600_)_2457T pBAD_)] ×100. Assays were completed with three biological replicates and repeated three times. Representative data are shown. Significance was calculated using a one-way ANOVA with *post hoc* Tukey HSD, *P* < 0.05. *, statistically significant compared to wild type. Complete statistical analysis is provided in Table S5.

Briefly, Congo red phenotypes (CR^+^ = red = virulent, and CR^−^ = white = avirulent) for each mutant were observed on Congo red plates under either non-inducing or inducing conditions (0.2% D-glucose or L-arabinose, respectively). Under non-inducing conditions, all colonies displayed a CR^−^ phenotype (Fig. S4). As expected, under inducing conditions, wild-type VirB appeared CR^+^ phenotype (Fig. 5A). In contrast, mutants found to be incapable of regulating the *icsP* promoter (I65A, T68A, F74A, R93A, and R94A), displayed a CR^−^ phenotype (Fig. 5A and B), similar to that seen under non-inducing conditions (Fig. S4). These findings are consistent with the idea that native residues at these positions are needed for *Shigella* virulence phenotypes. Notably, in these assays, VirB T68S and R95A exhibited CR^+^ phenotypes similar to wild type (Fig. 5A and B), whereas the annotated DNA-binding mutant K152E displayed an intermediate level of Congo red binding, statistically different from both the wild-type and those displaying a true CR^−^ phenotype. Collectively, these results show that key residues in VirB predicted to bind CTP are required for Congo red binding, a phenotype positively correlated with *Shigella* virulence.

### Key residues in the predicted CTP binding pocket are required for VirB focus formation *in vivo*

Members of the ParB superfamily, to which VirB belongs, show a discrete subcellular localization pattern within the bacterial cell during faithful plasmid/chromosome segregation, an activity associated with engaging its DNA recognition site (50, 51, 81 – 84). Even though VirB has an entirely different cellular role, a recent study in our lab discovered that VirB also forms discrete foci within *Shigella* cells, which are dependent upon VirB binding to its recognition site (85). We next investigated what effect VirB mutants would have on discrete focus formation when fused to superfolder green fluorescent protein (sfGFP) (86, 87). To test this, the 5′ end of five *virB* mutant alleles encoding substitutions in either T68 (both Ala and Ser), R93, R94, or R95, were introduced into the pBAD promoter located on the low-copy (~15–20 copies per cell) plasmid, pGB682. The resulting plasmids were induced in a *virB* mutant derivative of *Shigella flexneri* (AWY3) (85). The distribution of foci was quantified using the number of maxima detected per cell. Based on our previous study (85), any observed foci would likely be caused by the interaction of VirB with the large virulence plasmid.

**Fig 6:**
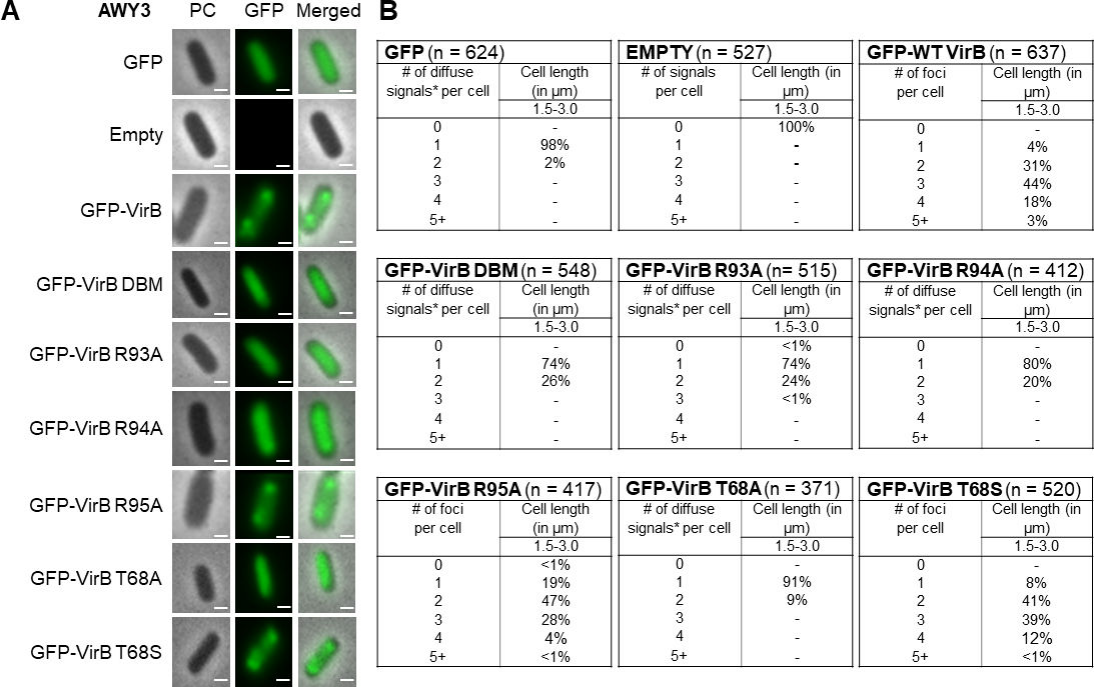
Key residues in the CTP binding pocket are required for VirB focus formation *in vivo*. (**A**) Live-cell imaging of GFP-VirB derivatives in a *virB* mutant strain of *S. flexneri* (AWY3) induced with 0.02% L-arabinose. Phase-contrast, PC, (left column), fluorescence (middle column) (GFP row, 28 ms exposure; GFP-VirB derivatives and empty rows, 98 ms exposure), and merged (right column). Bars represent 1 µM. (**B**) Quantification of fluorescent signals observed during live-cell imaging of GFP-VirB derivatives using MicrobeJ. Within tables, a hyphen indicates that no cells were found in this category in any of the images captured; *, maxima detected by MicrobeJ. Complete statistical analysis is provided in Table S6 and field-of-view images are found in Fig. S5.

As expected, in cells producing GFP only, there was a diffuse GFP signal, with 98% of cells having a single focus (maxima), whereas the empty plasmid control gave no GFP signal (Fig. 6). Cells producing GFP-VirB displayed discrete focus formation, with a majority of cells having 2–3 foci per cell, as described previously (85). This effect is likely dependent upon VirB engaging its DNA recognition site (Fig. 6), supported by a fusion carrying two substitutions in the DNA-binding domain (GFP-VirB DBM) exhibiting a diffuse signal like the GFP control (Fig. 6). When GFP was fused to VirB with alanine substitutions at key residues in the putative CTP binding pocket (R93A, R94A, or T68A), no foci were observed, also resembling the GFP control. In contrast, GFP-VirB R95A and T68S displayed discrete focus formation, with most cells forming two foci, similar to the wild-type fusion (Fig. 6). This is consistent with R95 being a non-conserved but proximal residue to the CTP binding pocket and T68S potentially restoring hydrogen bonding at this position. In sum, these findings demonstrate that R93, R94, and T68, residues in the CTP binding pocket, are required for VirB focus formation *in vivo*.

### Characterizing the order of VirB, CTP, and DNA binding site interactions

Our previous work had revealed that focus formation by GFP-VirB fusions was completely dependent upon the DNA binding activity of VirB *in vivo* (85). Consequently, the data described above (and shown in Fig. 6) raised the possibility that substitutions R93A, R94A, and T68A render the VirB protein incapable of binding to DNA. To further explore the DNA binding activity of our VirB mutants*,* we exploited a previously made *in vivo* DNA binding tool, known as pBT-P*icsP* (62). This genetic construct contains a VirB binding site positioned immediately adjacent to the −35 sequence of a constitutively active promoter, P*tac*. When VirB binds to its recognition site, it blocks transcription, likely through steric hindrance as RNA polymerase cannot access the −35 element (DNase I protection assays support this assertion [62]), providing a way to measure the binding activity of VirB *in vivo*.

**Fig 7:**
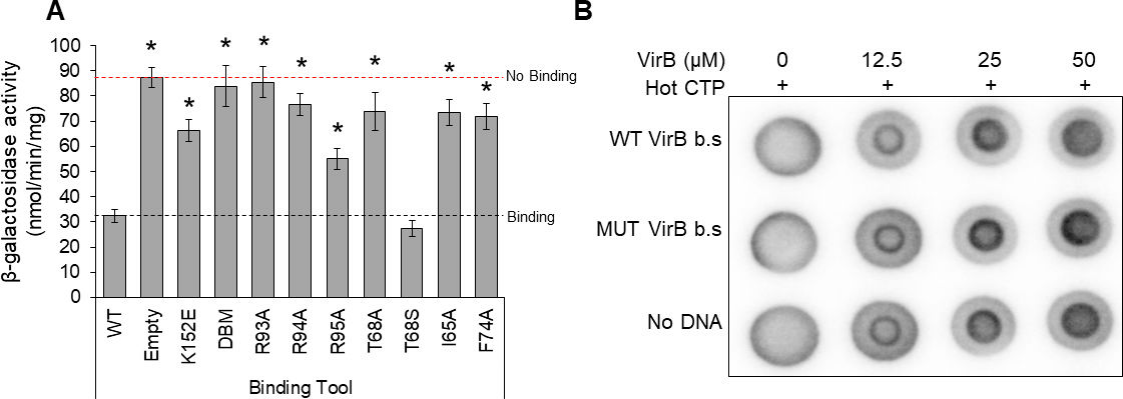
Characterizing the order of VirB-, CTP-, and DNA-binding site interactions. (**A**) P*tac* promoter activity of pBT-P*icsP* in the presence of pBAD-*virB* mutants in a *S. flexneri virB*::Tn5, as determined by β-galactosidase assays. Assays were completed with three biological replicates and repeated three times. Representative data are shown. Dash lines represent activities generated by the positive and negative controls. Significance was calculated using a one-way ANOVA with *post hoc* Tukey HSD, *P < 0.05. *, statistically significant compared to wild type. Complete statistical analysis provided in Table S7. Note, K152E and R95A are statistically different from both wild type and the empty plasmid control. (**B**) DRaCALA DNA dependency assay assessing the ability of VirB to bind CTP in the presence/absence of various DNA substrates. Three technical replicates were completed for each trial and three independent trials of this experiment were run. A representative technical replicate is shown.

pBT-P*icsP* was introduced into a *virB* mutant derivative of *Shigella* (AWY3) carrying different pBAD-*virB* derivatives, each encoding the VirB mutants described in this work. P*tac* activity was subsequently measured using β-galactosidase assays. As expected, wild-type VirB led to low levels of *Ptac* activity, consistent with it binding to its site and blocking transcription, whereas the empty plasmid control and a well-characterized DNA binding mutant (DBM) exhibited high levels of promoter activity, consistent with no interference (Fig. 7A). Interestingly, the VirB mutants with the following substitutions, R93A, R94A, T68A, I65A, and F74A, did not interfere with the activity of P*tac* (Fig. 7A), suggesting that these proteins do not engage the VirB recognition site. In contrast, VirB T68S blocked P*tac* activity like wild-type VirB (Fig. 7A), and curiously, VirB R95A and K152E showed intermediate phenotypes that were significantly different from wild-type and the double binding mutant (DBM) (Table S7). These data provide strong evidence that VirB proteins carrying substitutions in key residues of the predicted CTP binding pocket are indeed defective in DNA binding. These findings align perfectly with our preceding assays (Fig. 4 to 6) because the site-specific DNA binding activity of VirB is a requirement for each of the phenotypes that were tested.

Having established that CTP is likely needed for the DNA-binding activity of VirB *in vivo*, we next chose to investigate whether CTP binding by VirB is influenced by different DNA substrates. *C. vibriodes* ParB and *B. subtilis* Spo0J, two members of the classic ParB clade, do not bind CTP in the absence of their DNA recognition site, *parS* (60, 61). Consequently, we wanted to know if VirB requires its cognate site in order to bind CTP. To do this, we returned to DRaCALA assays. Purified VirB protein was incubated, at various concentrations, with radiolabeled (hot) α^32^P-CTP and, where appropriate, no DNA or DNA containing either a wild-type or mutated VirB-binding site was added (Fig. 7B). Irrespective of whether DNA was present or not, CTP binding by VirB remained unchanged, revealing that DNA is not a requirement for CTP binding by VirB. This finding again highlights differences between VirB and members of the classic ParB clade, and collectively these last two studies provide new mechanistic insight into the VirB regulatory protein and its requirements for, and interactions with, CTP and its DNA recognition site.

## DISCUSSION

In this work, we focus on the transcriptional anti-silencing protein, VirB, which functions to offset H-NS-mediated silencing of *Shigella* virulence genes on the large virulence plasmid. We show that VirB is a member of a fast-evolving clade of ParB proteins that is distinct from the slow-evolving versions that function in the segregation of the primary cellular chromosome (Fig. 1). Using two different approaches (ITC and DRaCALA), we show that, like members of the slow-evolving clade, VirB binds a highly unusual ligand, CTP, preferentially and with specificity (Fig. 2 and 3). VirB residues likely required for CTP binding were identified using amino acid alignments to the best-characterized members of the ParB family and were shown to be necessary for (i) VirB-dependent anti-silencing of virulence genes, (ii) generation of a Congo red positive phenotype, which correlates with *Shigella* virulence, and (iii) the ability of the VirB protein to form discrete foci when fused to GFP (Fig. 4 to 6). Subsequent studies revealed that these same VirB residues are necessary for VirB to engage its DNA binding site *in vivo* and that VirB binds CTP equivalently regardless of whether DNA bearing its cognate site is present or not (Fig. 7). Consequently, our work provides a new perspective on the ParB superfamily, is the first to link virulence attributes of *Shigella* to the highly unusual nucleoside triphosphate ligand, CTP and begins to reveal the role that CTP and the VirB binding site play in the VirB-dependent mechanism of virulence gene regulation.

To the best of our knowledge, the phylogenetic analysis of the ParB superfamily, included in this work, is the first to include VirB. Our analysis reveals that this superfamily is comprised of two distinct clades, one that contains the classic ParBs and a second, fast-evolving clade, containing *Shigella* VirB (Fig. 1 and Fig. S1). The latter is primarily comprised of plasmid-encoded proteins found in Proteobacteria. Our studies highlight that at least two members of this fast-evolving clade have become separated, in terms of their gene-neighborhoods, from *parA* (which encodes the ATPase that functions in faithful DNA segregation), i.e., *M. xanthus* PadC (*padC* is the fourth gene in an operon encoding bactofilins [88]) and *S. flexneri* VirB (*virB* is monocistronic and located immediately after the *ipa* operon, which encodes type III effector proteins, on pINV [7, 8]) (Fig. 1; Fig. S1). While PadC of *M. xanthus* still interacts with a ParA protein, encoded by the *parA* linked to *M. xanthus parB* (88), our analysis reveals that VirB has lost the N-terminal arginines which mediate interactions between ParB and ParA in other members of the family (Fig. S2). For VirB, the loss of this ParA interaction domain is especially important because the virulence plasmid of *Shigella flexneri* is a mosaic of ancestral plasmids and possesses an intact and functioning ParAB/parS partitioning system as well as an StbAB system (47). As such, the loss of a functional connection to ParA has likely gone hand-in-hand with the acquisition of a new function (46, 89), allowing this protein to co-opt a new role as a transcription factor. It seems likely that the rapid evolution of members of the plasmid-borne clade may have allowed some of these proteins to adopt new functions based on their existing biochemistry, i.e., NTP and DNA binding. Future investigations will test these hypotheses.

A major thrust of our research is to understand the molecular mechanism that is used by VirB to control *Shigella* virulence (12, 22, 24, 38, 62, 85). In pursuit of this, we have drawn from work on the ParB proteins and how they interface with DNA (52, 55, 73, 82, 90, 91). VirB and ParB proteins engage DNA as homodimeric proteins, through the recognition of a similar inverted repeat (20, 40, 41, 48, 92). Recently, though members of the ParB family (*B. subtilis* Spo0J [60], *C. vibriodes* ParB [61], and *M. xanthus* ParB [59]) have been identified to bind and hydrolyze a unique ligand, the nucleoside triphosphate CTP, in order to switch between open and closed clamp conformations while spreading along DNA (59 – 61). Here, we report that *Shigella* VirB binds CTP preferentially and with specificity (Fig. 2 and 3), with binding affinities (K_D_) ranging from 9.3 to 14.3 µM. These K_D_ values are similar to those calculated for *B. subtilis* Spo0J (60) and below those calculated for *M. xanthus* ParB (59). Based on amino acid sequence alignments and protein structural analyses, there appears to be only a single CTP-binding pocket per VirB monomer. Thus, it is likely that the binding stoichiometry of VirB (monomer) to CTP is 1:1. These extrapolations are supported by the ParB literature where the ratio of ParB (monomer) to CTP is also 1:1 (59). Our work demonstrates that VirB is a bona fide CTP-binding protein and only the second protein within the fast-evolving clade characterized to bind CTP.

Having established that VirB binds CTP, subsequent alignments with ParB proteins that bind CTP allowed key residues, predicted to lie in the CTP binding pocket of VirB, to be identified. These were located in two separate regions at the N-terminus of VirB (Fig. 4A). Residues I65, T68, and F74 (Box I residues [59]) are predicted to directly interact with the cytidine base, whereas G91, R93, and R94 (Box II residues [59]) are predicted to interact with the triphosphate motif of CTP (59 – 61). In ParB, substitutions of equivalent residues in these two regions led to a direct loss of CTP binding (80, 93) and prevented ParB from undergoing a closed clamp conformation change. These activities are necessary for proper partitioning of the DNA (80, 93). In this work, we show that substitution of equivalent residues in VirB leads to a loss of transcriptional anti-silencing activity at the well-characterized VirB-dependent promoter *icsP* (Fig. 4B), a loss of the Congo red phenotype, which strongly correlates with *Shigella* virulence (Fig. 5) (16), and a loss of discrete focus formation in the *Shigella* cytoplasm when VirB mutants were fused to GFP (Fig. 6). Since focus formation of GFP-VirB is wholly dependent on VirB engaging its recognition site *in vivo*, the results of our phenotypic studies suggested that our cohort of VirB mutants, while stably produced, may have been defective in forming normal VirB-DNA interactions. Subsequent assays validated this finding, revealing that VirB mutants carrying substitutions in key residues of the CTP binding pocket were defective in engaging their DNA recognition site *in vivo* (Fig. 7A). As such, these findings, along with our DNA dependency DRaCALAs (Fig. 7B), which show that DNA is not a requirement for CTP binding by VirB, begin to reveal the interplay between the tripartite regulatory system involving VirB, CTP, and its DNA recognition site. Future work will expand upon these findings to gain further mechanistic insight into how VirB, CTP, and DNA interactions underpin the process of transcriptional anti-silencing of virulence genes in the pathogen *Shigella*.

Interestingly, no evidence of CTP hydrolysis was observed by VirB in our ITC experiments, although this is an activity demonstrated by the classic ParBs (59 – 61). An absence of CTP hydrolysis was shown for *M. xanthus* PadC (59), another member of the fast-evolving clade. While two key residues required for hydrolysis, Q127 and K130, are found in VirB (in the so-called Box III, where they are needed for metal coordination and binding the α-phosphate of the NTP, respectively), several members of the fast-evolving clade, including VirB, have a substitution at another highly conserved residue in this region (E to I at position 126 in VirB, which coordinates one of the divalent metal ions needed for CTP hydrolysis). The substitution of this highly conserved acidic residue with a hydrophobic residue could potentially diminish CTPase activity while still supporting CTP-binding (as the other two residues Q127 and K130 are present). This is intriguing for multiple reasons. First, the current ParB model implicates CTP hydrolysis in the reopening of the sliding clamp, thus playing a critical role in the recycling of ParB bound DNA back to the cytoplasmic pool (59 – 61, 74). Second, the elimination of CTPase activity results in more diffuse ParB-dependent foci in the cytoplasm and defects in chromosome segregation (93, 94), likely due to the inability of ParB to recruit other ParB molecules to distal DNA (95). Hence, the potential lack of CTP hydrolysis in VirB raises the question of how VirB proteins are released from DNA and how VirB forms tight foci without CTPase activity. It is of note that our ITC experiments are performed in the absence of DNA; therefore, CTP hydrolysis may only be triggered when DNA is present. Work is currently ongoing to address whether VirB is capable of hydrolyzing CTP and whether this activity depends on the presence of DNA.

To conclude, this work provides novel insight into the ParB superfamily through the discovery that there are two distinct clades, the classic ParBs and the fast-evolving clade (Fig. 1 and Fig. S1). We are the first to demonstrate that *Shigella* VirB is a bona fide CTP-binding protein (Fig. 2 and 3). With striking differences in both homology and function, VirB is the first plasmid-borne ParB family member shown to bind CTP for its role as a virulence gene regulator. Our findings raise the possibility that other functionally distinct members of the fast-evolving clade may also bind CTP, therefore broadening our understanding of the ParB superfamily, a group of bacterial proteins that play critical roles in many different bacteria. Lastly, our finding that key residues in the predicted CTP binding pocket of VirB, are required for the virulence attributes of *Shigella* (Fig. 4 to 6), implicates CTP as an essential ligand for virulence gene regulation in *Shigella* and raises the possibility that virulence may be linked to fluctuations in cellular CTP pools. Future studies that extend this work are clearly needed. These studies will probe how CTP mechanistically influences transcriptional anti-silencing by VirB and delineate the role CTP plays in the control of *Shigella* virulence.

## MATERIALS AND METHODS

### Sequence, structure, and phylogenetic analysis

PSIBLAST searches (96) were used to identify homologs of VirB from the NCBI NR database clustered at 90% sequence identity (NR90) by the MMSEQS program (97). To reduce redundancy, the sequences were further clustered with variable length and score thresholds of 0.7 and 0.3, respectively. A multiple sequence alignment was generated using the Mafft program (98) with a local pair algorithm, maxiterate set to 3,000, op set to 1.2, and ep set to 0.5.

Secondary structures were predicted using the JPred program (99). Structures were rendered, compared, and superimposed using the Mol* program. Structural models were generated using Alphafold2 program, with templates obtained from HHpred searches for the neural networks deployed by the program.

Phylogenetic analysis was conducted using the maximum likelihood method implemented in the IQtree program (RRID: SCR_017254) (100) with a mixed model combining the Q.pfam substitution matrix, a discrete gamma-distributed model with four rate categories and one invariant category, and a FreeRate model with four rate categories. Bootstrap values were calculated using the ultrafast bootstrap method with 1,000 replicates. Phylogenetic trees were rendered using the FigTree program (RRID: SCR_008515) (http://tree.bio.ed.ac.uk/software/figtree/) and rooted via the midpoint rooting. Similar clades were also obtained when the tree was rooted with distant ParB domains such as plant sulfuredoxins or bacteriophage ParB-like proteins. The differential evolutionary rate plots were calculated using a matrix containing inter-sequence log-corrected pairwise maximum-likelihood distances computed using the FastTree program (101). The distances were centered and scaled using the min-max method and then plotted as Kernel density to normalize the overall area under each curve to 1. A two-sample *t*-test was used to estimate the significance of the difference between the mean values of the fast-evolving and the slow-evolving clades. All computations were performed using the R language.

### Bacterial strains, plasmids, and media

The strains and plasmids used in this study are listed in Table S1. *S. flexneri* strains were routinely grown on trypticase soy agar (TSA; trypticase soy broth [TSB] containing 1.5% [wt/vol] agar). When appropriate, maintenance of the virulence plasmid was examined by Congo red binding on TSA plates containing 0.01% (wt/vol) Congo red. Depending on the assay, liquid cultures were grown overnight at 30°C in LB broth or minimal media, which limits autofluorescence during imaging (M9 minimal medium supplemented with 0.4% D-glucose, 0.4% Casamino Acids, 0.01 mg/mL nicotinic acid, 0.01 mg/mL tryptophan, 0.01 mg/mL thiamine, 0.1 mM CaCl_2_, and 0.5 mM MgSO_4_) (102, 103). Overnight cultures were diluted 1:100 and subcultured at 37°C with aeration in the specified media (40 mM glycerol replaced d-glucose in minimal medium to allow induction of pBAD to occur [104]). Where necessary, diluted cultures were induced with 0.2% or 0.02% L-arabinose for 3 hours of a 5-hour growth period. To ensure plasmid maintenance, antibiotics were used at the following final concentrations: ampicillin, 100 µg mL^−1^, and chloramphenicol, 25 µg mL^−1^.

### Construction of *virB* mutant alleles

*virB* mutant alleles were created using megaprimer PCR (primers found in Table S2) (105, 106). The resulting alleles were then introduced into pBAD18 using EcoRI and HindIII. Each mutant was diagnosed using EcoRV and verified by Sanger dideoxy sequencing. All constructs made are listed in Table S1 and the primers used are listed in Table S2.

### Construction of plasmids producing GFP-VirB fusions

The *gfp* gene used throughout this work encodes a superfolder GFP (sfGFP) (86, 87). GFP-VirB mutant fusions were produced from pBAD-*sfgfp-linker-virB* constructs (85). In the wild-type *virB* coding region of pJNS12 (85), an XhoI site is found at the 5′ end, and a HindIII restriction site is found after the stop codon. Thus, *virB* derivatives (sourced from pATM324 derivatives) were introduced into pJNS12 using these restriction sites to create pBAD-*sfgfp-linker-virB* mutant derivatives (pTMG19-21, and pAMO18-19). Each fusion allele was verified by Sanger dideoxy sequencing.

### Construction of pBT-P*icsP* (Cm^r^)

This *in vivo* binding tool was modified from the original pBT-*PicsP*, which conferred ampicillin resistance (24). For this work, the *cat* promoter and gene was inserted into pBT-P*icsP* (Amp^r^) to replace the *bla* gene. The resulting plasmid was verified by NcoI digest and its ability to confer chloramphenicol resistance.

### VirB protein purification

VirB was purified by the Monserate Biotechnology Group (San Diego, CA) using a protocol described previously (38).

### Isothermal Titration Calorimetry

The ITC instrument (MicroCal PEAQ-ITC, Malvern Instruments, Worcestershire, United Kingdom) was used at 25°C, with binding buffer consisting of 25 mM HEPES/NaOH, pH 7.6, 100 mM NaCl, 5 mM MgCl_2_, 0.1 mM EDTA, 0.5 mM β-mercaptoethanol, and 5% (vol/vol) glycerol. The measurement cell and injection syringe were washed thoroughly prior to each run. A 300 µL sample of purified VirB protein, at 45–90 μM, or buffer was used to load the measurement cell (200 µL analytical volume). The injection syringe was filled with buffer containing 3 mM of nucleotides as indicated (CTPγS, CTP, or UTP). A single 0.4 µL priming injection was followed by twelve 3.0 µL injections at 150 seconds intervals. Data were plotted and analyzed using MicroCal PEAQ-ITC Analysis Software to obtain K_D_ values.

### Differential radial capillary action of ligand assay

Differential radial capillary action of ligand assay, DRaCALA, was adapted from references (59 – 61, 79). For competition assays, radiolabeled (hot) α^32^P-CTP (5 nM final) and unlabeled (cold) nucleotides (500 µM final) were prepared in buffer containing 500 mM NaCl, 500 mM Tris/HCl pH 7.5, 50 mM MgCl_2_, and kept on ice. Purified VirB protein (50 µM final) was added to each reaction and incubated for 10 minutes at room temperature. For DNA dependency assays, radiolabeled (hot) α^32^P-CTP (5 nM final) was prepared in buffer containing 500 mM NaCl, 500 mM Tris/HCl pH 7.5, 50 mM MgCl_2_, and kept on ice. Purified VirB protein (50 µM final) and various DNA substrates (50 ng) were added to each reaction and incubated for 10 minutes at room temperature. After incubation, 4 µL were spotted onto dry nitrocellulose (GE Healthcare) in technical replicates of three, allowed to air dry, and then exposed to a storage phosphor screen (Kodak). The screen was imaged on an Amersham Typhoon. Ligand binding was indicated by a darker inner core staining due to rapid immobilization of the protein and was quantified by densitometric analysis of the inner and outer core of each spot using AzureSpot Analysis Software version 2.0.062. The “Analysis Toolbox” was used to draw a circle around the outer and inner core of each spot. Circles were copied using the “duplicate” feature and dragged to adjacent spots so that direct comparisons could be made. The densitometric output for each spot was used to calculate the fraction bound (F_B_) using the following equation: FB = (I_inner_ − {A_inner_ × [(I_total_ − I_inner_) / (A_total_ − A_inner_)]})/I_total_ (79).

### Quantification of transcriptional anti-silencing by VirB derivatives

To quantify the ability of VirB derivatives to regulate transcription, promoter activities of P*icsP-lacZ* (pAFW04) were measured using β-galactosidase assays. Briefly, overnight cultures were diluted in LB containing appropriate antibiotics (ampicillin, 100 µg mL^−1^, or chloramphenicol, 25 µg mL^−1^) and induced, as described previously, these cultures were then lysed and β-galactosidase activity was measured using a modified Miller protocol (12, 103).

### Quantification of protein levels of VirB derivatives

Protein levels of VirB mutants were analyzed by Western Blot analysis, as described in (12, 85). After growth, cells were normalized to cell density (OD_600_), harvested, and washed with 1 mL 0.2 M Tris buffer (pH 8.0). Cells were resuspended in 200 µL 10 mM Tris (pH 7.4), and 50 µL 5X SDS-PAGE buffer. Equal volumes of normalized and heat-denatured protein preparations were electrophoresed on 12.5% SDS-PAGE gels alongside a SeeBlue Plus2 Prestained Standard (Invitrogen). VirB was detected using an affinity-purified anti-VirB antibody obtained from Pacific Immunology. A GE anti-rabbit IgG-horseradish peroxidase (HRP; NA9340) secondary antibody was used. All blots were imaged using “auto expose” with chemiluminescence detection (Azure c400; Azure Biosystems).

### Quantification of Congo red binding

Congo red binding of each VirB mutant was quantified using the protocol described in reference (104). Briefly, AWY3 (*virB::*Tn5) harboring pBAD, pBAD-*virB,* or pBAD-*virB* mutants were grown overnight in LB supplemented with 0.2% (wt/vol) D-glucose. The following day, cultures were serially diluted (100-fold dilutions) and ~6 µL of each dilution was spotted onto TSA Congo red plates supplemented with either 0.2% (wt/vol) L-arabinose or D-glucose. Plates were incubated overnight at 37°C. Phenotypes were imaged using the auto-exposure setting with a resolution set at 120 µm (Azure 400; Azure Biosystems) using visible light and RGB detection [captured with Cy2 (blue)]. The relative amount of Congo red dye bound by the cells was quantified by resuspending three culture spots in 750 µL of 25% ethanol for each sample. Optical densities of cell suspensions were measured at OD_600_, prior to centrifugation. The OD_498_ of the supernatant was then measured to quantify the amount of Congo red released from cells and normalized to cell density. Relative Congo red bound by cells was calculated using the following equation: [(OD_498_/OD_600_)/(average (OD_498_/OD_600_)_2457T pBAD_)] × 100.

### Visualization of GFP fusion proteins and quantification of foci

Visualization of GFP fusion proteins and quantification of foci were determined by fluorescence microscopy using the protocol described in reference 85 with the following exceptions: fluorescence excitation was performed (X-cite 120LED) at a range of 470–525 nm using 50%–75% exposure for all captured images and 4–5 fields of view were captured at random for each experimental strain, with roughly 60 cells per field of view. Statistical analyses were performed to compare the distributions of foci as described in statistical analyses.

### *In vivo* DNA binding assay

To determine if key residues in the predicted CTP binding pocket are required for VirB:DNA interactions, an *in vivo* binding assays was used (62). pBT-P*icsP* (Cm^r^; pJNS22) was introduced into the *S. flexneri virB* mutant strain AWY3 carrying the L-arabinose inducible pBAD-*virB* (pATM324) or derivatives. Cultures were grown overnight (16 hours) at 30°C in LB broth containing 0.2% D-glucose, ampicillin (100 µg mL^−1^), and chloramphenicol (25 µg mL^−1^). Prior to cell lysis, overnight cultures were diluted 1:100 and grown for 8 hours at 37°C with aeration (325 rpm in a LabLine/Barnstead 4000 MaxQ shaker) in LB broth containing 0.2% L-arabinose, ampicillin (100 µg mL^−1^), and chloramphenicol (25 µg mL^−1^). Promoter activities were determined by measuring β-galactosidase activity (protocol adapted from reference 103 and described in reference 12).

### Statistical analyses

Statistical calculations were performed using IBM SPSS Statistics for Windows, version 28.01.0. One-way ANOVA tests were routinely used and *post hoc* analyses were performed as indicated in figure legends. Kolmogorov-Smirnov test was used to assess statistical differences amongst distributions of foci in VirB mutants. Throughout this work, statistical significance is represented as, * indicating a *P* < 0.05.

## SUPPLEMENTAL MATERIAL

Supplemental material part 1 mbio.01519-23-s0001.pdf Table of contents, Tables S1-S7, and Figure S1.

Supplemental material part 2 mbio.01519-23-s0002.pdf Figures S2-S5 and references.

ASM does not own the copyrights to Supplemental Material that may be linked to, or accessed through, an article. The authors have granted ASM a non-exclusive, world-wide license to publish the Supplemental Material files. Please contact the corresponding author directly for reuse.

## References

[1] Hawkins HP . 1909. The identity of British ulcerative colitis and tropical bacillary dysentery. Br Med J 2:1331–1332. doi:10.1136/bmj.2.2549.1331 20764728 PMC2321382

[2] Labrec EH , Schneider H , Magnani TJ , Formal SB . 1964. Epithelial cell penetration as an essential step in the pathogenesis of bacillary dysentery. J Bacteriol 88:1503–1518. doi:10.1128/jb.88.5.1503-1518.1964 16562000 PMC277436

[3] Niyogi SK . 2007. Increasing antimicrobial resistance--an emerging problem in the treatment of shigellosis. Clin Microbiol Infect 13:1141–1143. doi:10.1111/j.1469-0691.2007.01829.x 17953700

[4] Niyogi SK . 2005. Shigellosis. J Microbiol 43:133–143. 15880088

[5] Weatherspoon-Griffin N , Picker MA , Wing HJ . 2016. The genetic organization and transcriptional regulation of *Shigella* virulence genes, p 65–107. In Picking WD , WL Picking (ed), Shigella: Molecular and cellular biology. Caister Academic Press, UK. doi:10.21775/9781910190197

[6] Tobe T , Nagai S , Okada N , Adler B , Yoshikawa M , Sasakawa C . 1991. Temperature-regulated expression of invasion genes in Shigella flexneri is controlled through the transcriptional activation of the virB gene on the large plasmid. Mol Microbiol 5:887–893. doi:10.1111/j.1365-2958.1991.tb00762.x 1857209

[7] Watanabe H , Arakawa E , Ito K , Kato J , Nakamura A . 1990. Genetic analysis of an invasion region by use of a Tn3-lac transposon and identification of a second positive regulator gene, invE, for cell invasion of Shigella sonnei: significant homology of InvE with ParB of plasmid P1. J Bacteriol 172:619–629. doi:10.1128/jb.172.2.619-629.1990 1688841 PMC208485

[8] Adler B , Sasakawa C , Tobe T , Makino S , Komatsu K , Yoshikawa M . 1989. A dual transcriptional activation system for the 230 kb plasmid genes coding for virulence-associated antigens of Shigella flexneri. Mol Microbiol 3:627–635. doi:10.1111/j.1365-2958.1989.tb00210.x 2474742

[9] Hall CP , Jadeja NB , Sebeck N , Agaisse H . 2022. Characterization of MxiE- and H-NS-dependent expression of ipaH7.8, ospC1, yccE, and yfdF in Shigella flexneri. mSphere 7:e0048522. doi:10.1128/msphere.00485-22 36346241 PMC9769918

[10] Basta DW , Pew KL , Immak JA , Park HS , Picker MA , Wigley AF , Hensley CT , Pearson JS , Hartland EL , Wing HJ . 2013. Characterization of the ospZ promoter in Shigella flexneri and its regulation by VirB and H-NS. J Bacteriol 195:2562–2572. doi:10.1128/JB.00212-13 23543709 PMC3676053

[11] McKenna JA , Wing HJ . 2020. The antiactivator of type III secretion, OspD1, is transcriptionally regulated by VirB and H-NS from remote sequences in Shigella flexneri. J Bacteriol 202:e00072-20. doi:10.1128/JB.00072-20 32123035 PMC7186461

[12] Wing HJ , Yan AW , Goldman SR , Goldberg MB . 2004. Regulation of IcsP, the outer membrane protease of the Shigella actin tail assembly protein IcsA, by virulence plasmid regulators VirF and VirB. J Bacteriol 186:699–705. doi:10.1128/JB.186.3.699-705.2004 14729695 PMC321486

[13] Gall TL , Mavris M , Martino MC , Bernardini ML , Denamur E , Parsot C . 2005. Analysis of virulence plasmid gene expression defines three classes of effectors in the type III secretion system of Shigella flexneri. Microbiology (Reading) 151:951–962. doi:10.1099/mic.0.27639-0 15758240

[14] McKenna JA , Karney MMA , Chan DK , Weatherspoon-Griffin N , Becerra Larios B , Pilonieta MC , Munson GP , Wing HJ . 2022. The AraC/XylS protein MxiE and its coregulator IpgC control a negative feedback loop in the transcriptional cascade that regulates type III secretion in Shigella flexneri. J Bacteriol 204:e0013722. doi:10.1128/jb.00137-22 35703565 PMC9295595

[15] Payne SM , Finkelstein RA . 1977. Growth on Congo red agar: possible means of identifying penicillin-resistant non-penicillinase-producing gonococci. J Clin Microbiol 6:534–535. doi:10.1128/jcm.6.5.534-535.1977 411807 PMC274812

[16] Maurelli AT , Blackmon B , Curtiss R . 1984. Loss of pigmentation in Shigella flexneri 2a is correlated with loss of virulence and virulence-associated plasmid. Infect Immun 43:397–401. doi:10.1128/iai.43.1.397-401.1984 6360906 PMC263440

[17] Sankaran K , Ramachandran V , Subrahmanyam YV , Rajarathnam S , Elango S , Roy RK . 1989. Congo red-mediated regulation of levels of Shigella flexneri 2a membrane proteins. Infect Immun 57:2364–2371. doi:10.1128/iai.57.8.2364-2371.1989 2663721 PMC313456

[18] Meitert T , Pencu E , Ciudin L , Tonciu M , Mihai I , Nicolescu S . 1991. Correlation between Congo red binding as virulence marker in Shigella species and Sereny test. Roum Arch Microbiol Immunol 50:45–52. 1802051

[19] Sasakawa C , Kamata K , Sakai T , Murayama SY , Makino S , Yoshikawa M . 1986. Molecular alteration of the 140-megadalton plasmid associated with loss of virulence and Congo red binding activity in Shigella flexneri. Infect Immun 51:470–475. doi:10.1128/iai.51.2.470-475.1986 3002985 PMC262355

[20] Turner EC , Dorman CJ . 2007. H-NS antagonism in Shigella flexneri by VirB, a virulence gene transcription regulator that is closely related to plasmid partition factors. J Bacteriol 189:3403–3413. doi:10.1128/JB.01813-06 17307842 PMC1855880

[21] Beloin C , Dorman CJ . 2003. An extended role for the nucleoid structuring protein H-NS in the virulence gene regulatory cascade of Shigella flexneri. Mol Microbiol 47:825–838. doi:10.1046/j.1365-2958.2003.03347.x 12535079

[22] Castellanos MI , Harrison DJ , Smith JM , Labahn SK , Levy KM , Wing HJ . 2009. VirB alleviates H-NS repression of the icsP promoter in Shigella flexneri from sites more than one kilobase upstream of the transcription start site. J Bacteriol 191:4047–4050. doi:10.1128/JB.00313-09 19363111 PMC2698392

[23] Porter ME , Dorman CJ . 1997. Differential regulation of the plasmid-encoded genes in the Shigella flexneri virulence regulon. Mol Gen Genet 256:93–103. doi:10.1007/s004380050550 9349700

[24] Weatherspoon-Griffin N , Picker MA , Pew KL , Park HS , Ginete DR , Karney MM , Usufzy P , Castellanos MI , Duhart JC , Harrison DJ , Socea JN , Karabachev AD , Hensley CT , Howerton AJ , Ojeda-Daulo R , Immak JA , Wing HJ . 2018. Insights into transcriptional silencing and anti-silencing in Shigella flexneri: a detailed molecular analysis of the icsP virulence locus. Mol Microbiol 108:505–518. doi:10.1111/mmi.13932 29453862 PMC6311345

[25] Stoebel DM , Free A , Dorman CJ . 2008. Anti-silencing: overcoming H-NS-mediated repression of transcription in Gram-negative enteric bacteria. Microbiology (Reading) 154:2533–2545. doi:10.1099/mic.0.2008/020693-0 18757787

[26] Navarre WW , Porwollik S , Wang Y , McClelland M , Rosen H , Libby SJ , Fang FC . 2006. Selective silencing of foreign DNA with low GC content by the H-NS protein in Salmonella. Science 313:236–238. doi:10.1126/science.1128794 16763111

[27] Oshima T , Ishikawa S , Kurokawa K , Aiba H , Ogasawara N . 2006. Escherichia coli histone-like protein H-NS preferentially binds to horizontally acquired DNA in association with RNA polymerase. DNA Res 13:141–153. doi:10.1093/dnares/dsl009 17046956

[28] Williams RM , Rimsky S . 1997. Molecular aspects of the E. coli nucleoid protein, H-NS: a central controller of gene regulatory networks. FEMS Microbiol Lett 156:175–185. doi:10.1111/j.1574-6968.1997.tb12724.x 9513262

[29] Navarre WW , McClelland M , Libby SJ , Fang FC . 2007. Silencing of xenogeneic DNA by H-NS-facilitation of lateral gene transfer in bacteria by a defense system that recognizes foreign DNA. Genes Dev 21:1456–1471. doi:10.1101/gad.1543107 17575047

[30] Bouffartigues E , Buckle M , Badaut C , Travers A , Rimsky S . 2007. H-NS cooperative binding to high-affinity sites in a regulatory element results in transcriptional silencing. Nat Struct Mol Biol 14:441–448. doi:10.1038/nsmb1233 17435766

[31] Lucchini S , Rowley G , Goldberg MD , Hurd D , Harrison M , Hinton JCD . 2006. H-NS mediates the silencing of laterally acquired genes in bacteria. PLoS Pathog 2:e81. doi:10.1371/journal.ppat.0020081 16933988 PMC1550270

[32] Gordon BRG , Li Y , Cote A , Weirauch MT , Ding P , Hughes TR , Navarre WW , Xia B , Liu J . 2011. Structural basis for recognition of AT-rich DNA by unrelated xenogeneic silencing proteins. Proc Natl Acad Sci U S A 108:10690–10695. doi:10.1073/pnas.1102544108 21673140 PMC3127928

[33] Tendeng C , Badaut C , Krin E , Gounon P , Ngo S , Danchin A , Rimsky S , Bertin P . 2000. Isolation and characterization of vicH, encoding a new pleiotropic regulator in Vibrio cholerae. J Bacteriol 182:2026–2032. doi:10.1128/JB.182.7.2026-2032.2000 10715012 PMC101921

[34] Tendeng C , Bertin PN . 2003. H-NS in Gram-negative bacteria: a family of multifaceted proteins. Trends Microbiol 11:511–518. doi:10.1016/j.tim.2003.09.005 14607068

[35] Castang S , McManus HR , Turner KH , Dove SL . 2008. H-NS family members function coordinately in an opportunistic pathogen. Proc Natl Acad Sci U S A 105:18947–18952. doi:10.1073/pnas.0808215105 19028873 PMC2596223

[36] Ali SS , Soo J , Rao C , Leung AS , Ngai D-M , Ensminger AW , Navarre WW . 2014. Silencing by H-NS potentiated the evolution of Salmonella. PLoS Pathog 10:e1004500. doi:10.1371/journal.ppat.1004500 25375226 PMC4223078

[37] Colonna B , Casalino M , Fradiani PA , Zagaglia C , Naitza S , Leoni L , Prosseda G , Coppo A , Ghelardini P , Nicoletti M . 1995. H-NS regulation of virulence gene expression in enteroinvasive Escherichia coli harboring the virulence plasmid integrated into the host chromosome. J Bacteriol 177:4703–4712. doi:10.1128/jb.177.16.4703-4712.1995 7642498 PMC177236

[38] Picker MA , Karney MMA , Gerson TM , Karabachev AD , Duhart JC , McKenna JA , Wing HJ . 2023. Localized modulation of DNA supercoiling, triggered by the Shigella anti-silencer VirB, is sufficient to relieve H-NS-mediated silencing. Nucleic Acids Res 51:3679–3695. doi:10.1093/nar/gkad088 36794722 PMC10164555

[39] Gao X , Zou T , Mu Z , Qin B , Yang J , Waltersperger S , Wang M , Cui S , Jin Q . 2013. Structural insights into VirB-DNA complexes reveal mechanism of transcriptional activation of virulence genes. Nucleic Acids Res 41:10529–10541. doi:10.1093/nar/gkt748 23985969 PMC3905869

[40] Beloin C , McKenna S , Dorman CJ . 2002. Molecular dissection of VirB, a key regulator of the virulence cascade of Shigella flexneri. J Biol Chem 277:15333–15344. doi:10.1074/jbc.M111429200 11850420

[41] Taniya T , Mitobe J , Nakayama S , Mingshan Q , Okuda K , Watanabe H . 2003. Determination of the InvE binding site required for expression of IpaB of the Shigella sonnei virulence plasmid: involvement of a ParB boxA-like sequence. J Bacteriol 185:5158–5165. doi:10.1128/JB.185.17.5158-5165.2003 12923088 PMC181004

[42] Ireton K , Grossman AD . 1994. DNA-related conditions controlling the initiation of sporulation in Bacillus subtilis. Cell Mol Biol Res 40:193–198. 7874195

[43] Mohl DA , Easter J , Gober JW . 2001. The chromosome partitioning protein, ParB, is required for cytokinesis in Caulobacter crescentus. Mol Microbiol 42:741–755. doi:10.1046/j.1365-2958.2001.02643.x 11722739

[44] Jung A , Raßbach A , Pulpetta RL , van Teeseling MCF , Heinrich K , Sobetzko P , Serrania J , Becker A , Thanbichler M . 2019. Two-step chromosome segregation in the stalked budding bacterium Hyphomonas neptunium. Nat Commun 10:3290. doi:10.1038/s41467-019-11242-5 31337764 PMC6650430

[45] McLean TC , Le TB . 2023. CTP switches in ParABS-mediated bacterial chromosome segregation and beyond. Curr Opin Microbiol 73:102289. doi:10.1016/j.mib.2023.102289 36871427

[46] Buchrieser C , Glaser P , Rusniok C , Nedjari H , D’Hauteville H , Kunst F , Sansonetti P , Parsot C . 2000. The virulence plasmid pWR100 and the repertoire of proteins secreted by the type III secretion apparatus of Shigella flexneri. Mol Microbiol 38:760–771. doi:10.1046/j.1365-2958.2000.02179.x 11115111

[47] McVicker G , Hollingshead S , Pilla G , Tang CM . 2019. Maintenance of the virulence plasmid in Shigella flexneri is influenced by Lon and two functional partitioning systems. Mol Microbiol 111:1355–1366. doi:10.1111/mmi.14225 30767313 PMC6519299

[48] Lin DC , Grossman AD . 1998. Identification and characterization of a bacterial chromosome partitioning site. Cell 92:675–685. doi:10.1016/s0092-8674(00)81135-6 9506522

[49] Mohl DA , Gober JW . 1997. Cell cycle-dependent polar localization of chromosome partitioning proteins in Caulobacter crescentus. Cell 88:675–684. doi:10.1016/s0092-8674(00)81910-8 9054507

[50] Erdmann N , Petroff T , Funnell BE . 1999. Intracellular localization of P1 ParB protein depends on ParA and parS. Proc Natl Acad Sci U S A 96:14905–14910. doi:10.1073/pnas.96.26.14905 10611311 PMC24746

[51] Breier AM , Grossman AD . 2007. Whole-genome analysis of the chromosome partitioning and sporulation protein Spo0J (ParB) reveals spreading and origin-distal sites on the Bacillus subtilis chromosome. Mol Microbiol 64:703–718. doi:10.1111/j.1365-2958.2007.05690.x 17462018

[52] Murray H , Ferreira H , Errington J . 2006. The bacterial chromosome segregation protein Spo0J spreads along DNA from parS nucleation sites. Mol Microbiol 61:1352–1361. doi:10.1111/j.1365-2958.2006.05316.x 16925562

[53] Rodionov O , Lobocka M , Yarmolinsky M . 1999. Silencing of genes flanking the P1 plasmid centromere. Science 283:546–549. doi:10.1126/science.283.5401.546 9915704

[54] Rodionov O , Yarmolinsky M . 2004. Plasmid partitioning and the spreading of P1 partition protein ParB. Mol Microbiol 52:1215–1223. doi:10.1111/j.1365-2958.2004.04055.x 15130136

[55] Chen BW , Lin MH , Chu CH , Hsu CE , Sun YJ . 2015. Insights into ParB spreading from the complex structure of Spo0J and parS. Proc Natl Acad Sci U S A 112:6613–6618. doi:10.1073/pnas.1421927112 25964325 PMC4450421

[56] Fogel MA , Waldor MK . 2006. A dynamic, mitotic-like mechanism for bacterial chromosome segregation. Genes Dev 20:3269–3282. doi:10.1101/gad.1496506 17158745 PMC1686604

[57] Vecchiarelli AG , Neuman KC , Mizuuchi K . 2014. A propagating ATPase gradient drives transport of surface-confined cellular cargo. Proc Natl Acad Sci U S A 111:4880–4885. doi:10.1073/pnas.1401025111 24567408 PMC3977271

[58] Lim HC , Surovtsev IV , Beltran BG , Huang F , Bewersdorf J , Jacobs-Wagner C . 2014. Evidence for a DNA-relay mechanism in ParABS-mediated chromosome segregation. Elife 3:e02758. doi:10.7554/eLife.02758 24859756 PMC4067530

[59] Osorio-Valeriano M , Altegoer F , Steinchen W , Urban S , Liu Y , Bange G , Thanbichler M . 2019. ParB-type DNA segregation proteins are CTP-dependent molecular switches. Cell 179:1512–1524. doi:10.1016/j.cell.2019.11.015 31835030

[60] Soh Y-M , Davidson IF , Zamuner S , Basquin J , Bock FP , Taschner M , Veening J-W , De Los Rios P , Peters J-M , Gruber S . 2019. Self-organization of parS centromeres by the ParB CTP hydrolase. Science 366:1129–1133. doi:10.1126/science.aay3965 31649139 PMC6927813

[61] Jalal AS , Tran NT , Le TB . 2020. ParB spreading on DNA requires cytidine triphosphate in vitro. Elife 9:e53515. doi:10.7554/eLife.53515 32077854 PMC7053999

[62] Karney MM , McKenna JA , Weatherspoon-Griffin N , Karabachev AD , Millar ME , Potocek EA , Wing HJ . 2019. Investigating the DNA-binding site for VirB, a key transcriptional regulator of Shigella virulence genes, using an in vivo binding tool. Genes (Basel) 10:149. doi:10.3390/genes10020149 30781432 PMC6410309

[63] McKenna S , Beloin C , Dorman CJ . 2003. In vitro DNA-binding properties of VirB, the Shigella flexneri virulence regulatory protein. FEBS Lett 545:183–187. doi:10.1016/s0014-5793(03)00524-6 12804772

[64] Tobe T , Yoshikawa M , Mizuno T , Sasakawa C . 1993. Transcriptional control of the invasion regulatory gene virB of Shigella flexneri: activation by virF and repression by H-NS. J Bacteriol 175:6142–6149. doi:10.1128/jb.175.19.6142-6149.1993 7691791 PMC206708

[65] Schumacher MA , Mansoor A , Funnell BE . 2007. Structure of a four-way bridged ParB-DNA complex provides insight into P1 segrosome assembly. J Biol Chem 282:10456–10464. doi:10.1074/jbc.M610603200 17293348

[66] Shi R , Villarroya M , Ruiz-Partida R , Li Y , Proteau A , Prado S , Moukadiri I , Benítez-Páez A , Lomas R , Wagner J , Matte A , Velázquez-Campoy A , Armengod M-E , Cygler M . 2009. Structure-function analysis of Escherichia coli MnmG (GidA), a highly conserved tRNA-modifying enzyme. J Bacteriol 191:7614–7619. doi:10.1128/JB.00650-09 19801413 PMC2786596

[67] Brégeon D , Colot V , Radman M , Taddei F . 2001. Translational misreading: a tRNA modification counteracts a +2 ribosomal frameshift. Genes Dev 15:2295–2306. doi:10.1101/gad.207701 11544186 PMC312767

[68] Mikheil DM , Shippy DC , Eakley NM , Okwumabua OE , Fadl AA . 2012. Deletion of gene encoding methyltransferase (gidB) confers high-level antimicrobial resistance in Salmonella. J Antibiot (Tokyo) 65:185–192. doi:10.1038/ja.2012.5 22318332

[69] Perdigão J , Macedo R , Machado D , Silva C , Jordão L , Couto I , Viveiros M , Portugal I . 2014. GidB mutation as a phylogenetic marker for Q1 cluster Mycobacterium tuberculosis isolates and intermediate-level streptomycin resistance determinant in Lisbon, Portugal. Clin Microbiol Infect 20:278–284. doi:10.1111/1469-0691.12392 24102832

[70] Blattner FR , Plunkett G , Bloch CA , Perna NT , Burland V , Riley M , Collado-Vides J , Glasner JD , Rode CK , Mayhew GF , Gregor J , Davis NW , Kirkpatrick HA , Goeden MA , Rose DJ , Mau B , Shao Y . 1997. The complete genome sequence of Escherichia coli K-12. Science 277:1453–1462. doi:10.1126/science.277.5331.1453 9278503

[71] Sääf A , Monné M , de Gier JW , von Heijne G . 1998. Membrane topology of the 60-kDa Oxa1p homologue from Escherichia coli. J Biol Chem 273:30415–30418. doi:10.1074/jbc.273.46.30415 9804807

[72] Ulrych A , Holečková N , Goldová J , Doubravová L , Benada O , Kofroňová O , Halada P , Branny P . 2016. Characterization of pneumococcal Ser/Thr protein phosphatase phpP mutant and identification of a novel PhpP substrate, putative RNA binding protein Jag. BMC Microbiol 16:247. doi:10.1186/s12866-016-0865-6 27776484 PMC5078927

[73] Bartosik AA , Lasocki K , Mierzejewska J , Thomas CM , Jagura-Burdzy G . 2004. ParB of Pseudomonas aeruginosa: interactions with its partner ParA and its target parS and specific effects on bacterial growth. J Bacteriol 186:6983–6998. doi:10.1128/JB.186.20.6983-6998.2004 15466051 PMC522188

[74] Taylor JA , Seol Y , Budhathoki J , Neuman KC , Mizuuchi K . 2021. CTP and parS coordinate ParB partition complex dynamics and ParA-ATPase activation for ParABS-mediated DNA partitioning. Elife 10:e65651. doi:10.7554/eLife.65651 34286695 PMC8357417

[75] Kühn J , Briegel A , Mörschel E , Kahnt J , Leser K , Wick S , Jensen GJ , Thanbichler M . 2010. Bactofilins, a ubiquitous class of cytoskeletal proteins mediating polar localization of a cell wall synthase in Caulobacter crescentus. EMBO J 29:327–339. doi:10.1038/emboj.2009.358 19959992 PMC2824468

[76] Vasa S , Lin L , Shi C , Habenstein B , Riedel D , Kühn J , Thanbichler M , Lange A . 2015. β-Helical architecture of cytoskeletal bactofilin filaments revealed by solid-state NMR. Proc Natl Acad Sci U S A 112:E127–E136. doi:10.1073/pnas.1418450112 25550503 PMC4299214

[77] Shi C , Fricke P , Lin L , Chevelkov V , Wegstroth M , Giller K , Becker S , Thanbichler M , Lange A . 2015. Atomic-resolution structure of cytoskeletal bactofilin by solid-state NMR. Sci Adv 1:e1501087. doi:10.1126/sciadv.1501087 26665178 PMC4672760

[78] Koch MK , McHugh CA , Hoiczyk E . 2011. BacM, an N-terminally processed bactofilin of Myxococcus xanthus, is crucial for proper cell shape. Mol Microbiol 80:1031–1051. doi:10.1111/j.1365-2958.2011.07629.x 21414039 PMC3091990

[79] Roelofs KG , Wang J , Sintim HO , Lee VT . 2011. Differential radial capillary action of ligand assay for high-throughput detection of protein-metabolite interactions. Proc Natl Acad Sci U S A 108:15528–15533. doi:10.1073/pnas.1018949108 21876132 PMC3174574

[80] Jalal AS , Tran NT , Stevenson CE , Chimthanawala A , Badrinarayanan A , Lawson DM , Le TB . 2021. A CTP-dependent gating mechanism enables ParB spreading on DNA. Elife 10:e69676. doi:10.7554/eLife.69676 34397383 PMC8367383

[81] Sengupta M , Nielsen HJ , Youngren B , Austin S . 2010. P1 plasmid segregation: accurate redistribution by dynamic plasmid pairing and separation. J Bacteriol 192:1175–1183. doi:10.1128/JB.01245-09 19897644 PMC2820840

[82] Kusiak M , Gapczynska A , Plochocka D , Thomas CM , Jagura-Burdzy G . 2011. Binding and spreading of ParB on DNA determine its biological function in Pseudomonas aeruginosa. J Bacteriol 193:3342–3355. doi:10.1128/JB.00328-11 21531806 PMC3133298

[83] Donovan C , Schwaiger A , Krämer R , Bramkamp M . 2010. Subcellular localization and characterization of the ParAB system from Corynebacterium glutamicum. J Bacteriol 192:3441–3451. doi:10.1128/JB.00214-10 20435732 PMC2897671

[84] Broedersz CP , Wang X , Meir Y , Loparo JJ , Rudner DZ , Wingreen NS . 2014. Condensation and localization of the partitioning protein ParB on the bacterial chromosome. Proc Natl Acad Sci U S A 111:8809–8814. doi:10.1073/pnas.1402529111 24927534 PMC4066521

[85] Socea JN , Bowman GR , Wing HJ . 2021. VirB, a key transcriptional regulator of virulence plasmid genes in Shigella flexneri, forms DNA-binding site-dependent foci in the bacterial cytoplasm. J Bacteriol 203:e00627-20. doi:10.1128/JB.00627-20 33722845 PMC8117518

[86] Landgraf D , Okumus B , Chien P , Baker TA , Paulsson J . 2012. Segregation of molecules at cell division reveals native protein localization. Nat Methods 9:480–482. doi:10.1038/nmeth.1955 22484850 PMC3779060

[87] Pédelacq J-D , Cabantous S , Tran T , Terwilliger TC , Waldo GS . 2006. Engineering and characterization of a superfolder green fluorescent protein. Nat Biotechnol 24:79–88. doi:10.1038/nbt1172 16369541

[88] Lin L , Osorio Valeriano M , Harms A , Søgaard-Andersen L , Thanbichler M . 2017. Bactofilin-mediated organization of the ParABS chromosome segregation system in Myxococcus xanthus. Nat Commun 8:1817. doi:10.1038/s41467-017-02015-z 29180656 PMC5703909

[89] Radnedge L , Davis MA , Youngren B , Austin SJ . 1997. Plasmid maintenance functions of the large virulence plasmid of Shigella flexneri. J Bacteriol 179:3670–3675. doi:10.1128/jb.179.11.3670-3675.1997 9171415 PMC179163

[90] Graham TGW , Wang X , Song D , Etson CM , van Oijen AM , Rudner DZ , Loparo JJ . 2014. ParB spreading requires DNA bridging. Genes Dev 28:1228–1238. doi:10.1101/gad.242206.114 24829297 PMC4052768

[91] Sanchez A , Cattoni DI , Walter JC , Rech J , Parmeggiani A , Nollmann M , Bouet JY . 2015. Stochastic self-assembly of ParB proteins builds the bacterial DNA segregation apparatus. Cell Syst 1:163–173. doi:10.1016/j.cels.2015.07.013 27135801

[92] Radnedge L , Davis MA , Austin SJ . 1996. P1 and P7 plasmid partition: ParB protein bound to its partition site makes a separate discriminator contact with the DNA that determines species specificity. EMBO J 15:1155–1162. doi:10.1002/j.1460-2075.1996.tb00454.x 8605886 PMC450014

[93] Osorio-Valeriano M , Altegoer F , Das CK , Steinchen W , Panis G , Connolley L , Giacomelli G , Feddersen H , Corrales-Guerrero L , Giammarinaro PI , Hanßmann J , Bramkamp M , Viollier PH , Murray S , Schäfer LV , Bange G , Thanbichler M . 2021. The CTPase activity of ParB determines the size and dynamics of prokaryotic DNA partition complexes. Mol Cell 81:3992–4007. doi:10.1016/j.molcel.2021.09.004 34562373

[94] Antar H , Soh Y-M , Zamuner S , Bock FP , Anchimiuk A , Rios PDL , Gruber S . 2021. Relief of ParB autoinhibition by parS DNA catalysis and recycling of ParB by CTP hydrolysis promote bacterial centromere assembly. Sci Adv 7:eabj2854. doi:10.1126/sciadv.abj2854 34613769 PMC8494293

[95] Tišma M , Panoukidou M , Antar H , Soh Y-M , Barth R , Pradhan B , Barth A , van der Torre J , Michieletto D , Gruber S , Dekker C . 2022. ParB proteins can bypass DNA-bound roadblocks via dimer-dimer recruitment. Sci Adv 8:eabn3299. doi:10.1126/sciadv.abn3299 35767606 PMC9242446

[96] Altschul SF , Madden TL , Schäffer AA , Zhang J , Zhang Z , Miller W , Lipman DJ . 1997. Gapped BLAST and PSI-BLAST: a new generation of protein database search programs. Nucleic Acids Res 25:3389–3402. doi:10.1093/nar/25.17.3389 9254694 PMC146917

[97] Hauser M , Steinegger M , Söding J . 2016. MMseqs software suite for fast and deep clustering and searching of large protein sequence sets. Bioinformatics 32:1323–1330. doi:10.1093/bioinformatics/btw006 26743509

[98] Katoh K , Misawa K , Kuma K , Miyata T . 2002. MAFFT: a novel method for rapid multiple sequence alignment based on fast Fourier transform. Nucleic Acids Res 30:3059–3066. doi:10.1093/nar/gkf436 12136088 PMC135756

[99] Harada M , Nishitani H , Koga K , Miura I , Kimura A . 1993. Comparative studies on the metabolism of new fluorinated pyrimidine drugs in the liver by in vivo 19F magnetic resonance spectroscopic observation. Jpn J Cancer Res 84:197–202. doi:10.1111/j.1349-7006.1993.tb02855.x 8463136 PMC5919136

[100] Minh BQ , Schmidt HA , Chernomor O , Schrempf D , Woodhams MD , von Haeseler A , Lanfear R , Teeling E . 2020. IQ-TREE 2: new models and efficient methods for phylogenetic inference in the genomic era. Mol Biol Evol 37:1530–1534. doi:10.1093/molbev/msaa131 32011700 PMC7182206

[101] Price MN , Dehal PS , Arkin AP . 2010. FastTree 2--approximately maximum-likelihood trees for large alignments. PLoS One 5:e9490. doi:10.1371/journal.pone.0009490 20224823 PMC2835736

[102] Brandon LD , Goldberg MB . 2001. Periplasmic transit and disulfide bond formation of the autotransported Shigella protein IcsA. J Bacteriol 183:951–958. doi:10.1128/JB.183.3.951-958.2001 11208794 PMC94963

[103] Miller JH . 1992. A short course in bacterial Genetics: a laboratory manual and Handbook for Escherichia coli and related bacteria. Cold Spring Harbor Laboratory Press, Plainview, N.Y.

[104] Weatherspoon-Griffin N , Wing HJ . 2016. Characterization of SlyA in Shigella flexneri identifies a novel role in virulence. Infect Immun 84:1073–1082. doi:10.1128/IAI.00806-15 26831468 PMC4807491

[105] Barik S . 1993. Site-directed mutagenesis by double polymerase chain reaction : megaprimer method. Methods Mol Biol 15:277–286. doi:10.1385/0-89603-244-2:277 21400286

[106] Barik S . 1995. Site-directed mutagenesis by double polymerase chain reaction. Mol Biotechnol 3:1–7. doi:10.1007/BF02821329 7606501

