# Supplementary material for: VirB, a key transcriptional regulator of Shigella virulence, requires a CTP ligand for its regulatory activities
Source: mBio. 2023 Sep 20;14(5):e01519-23. doi: 10.1128/mbio.01519-23 (PMC10653881; doi:10.1128/mbio.01519-23)
Supplement: Supplemental material part 1 — Table of contents, Tables S1-S7, and Figure S1. [file mbio.01519-23-s0001.pdf]

Supporting Information for:

CTP is a required ligand for the regulatory activities of VirB, a key transcriptional regulator of *Shigella* virulence

Taylor M. Gerson[1], Audrey M. Ott[1], Monika MA. Karney[1], Jillian N. Socea[1], Daren R. Ginete[1], Lakshminarayan M. Iyer[2], L. Aravind[2], Ronald K. Gary[3], and Helen J. Wing[1*]

Table of Contents

Table S1. Bacterial strains and plasmid used in this study 2

Table S2. Primers used in this study 3

Table S3. Complete statistics for DRaCALA competition assay 4

Table S4. Complete statistics for anti-silencing of P*icsP* 5

Table S5. Complete statistics Congo red binding assay 6

Table S6. Complete statistics for fluorescence microscopy distributions 7

Table S7. Complete statistics for *in vivo* DNA binding assay 8

Figure S1. Maximum likelihood tree of ParB protein sequences including the VirB-like fast-evolving sequences 9

Figure S2. Multiple sequence alignment of slow-evolving (classic) and fast-evolving (e.g., VirB) ParB proteins containing the catalytic ParB, HTH, tetrahelical and C-terminal domains 10-15

Figure S3. Isothermal Titration Calorimetry buffer-only controls 16

Figure S4. Congo red binding activity of VirB mutants 17

Figure S5. Live cell imaging of GFP-VirB mutants in a *virB* mutant strain of *S. flexneri* 18-20

References 21

Table S1. Bacterial strains and plasmids used in this study

Label	Description	Reference
**Strains**		
S. flexneri		
AWY3	2457T *virB*::Tn5; Kn^r	(1)
**Plasmids**		
pATM324	pBAD18-*virB*; Amp^r	(2)
pBAD18	Arabinose-inducible pBAD expression vector, *ori* pBR*;* Amp^r	(3)
pADK15	pBAD-*virB* K152E; Amp^r	(4)
pTMG24	pBAD-*virB* K152E-R167E; Amp^r	This work
pTMG25	pBAD-*virB* G91S; Amp^r	This work
pDRG03	pBAD-*virB* R93A; Amp^r	This work
pDRG04	pBAD-*virB* R94A; Amp^r	This work
pDRG05	pBAD-*virB* R95A; Amp^r	This work
pAMO12	pBAD-*virB* T68A; Amp^r	This work
pAMO13	pBAD-*virB* T68S; Amp^r	This work
pAMO14	pBAD-*virB* I65A; Amp^r	This work
pAMO15	pBAD-*virB* F74A; Amp^r	This work
pAFW04	pACYC184 carrying WT *virB* in P*icsP*-*lacZ*; Cm^r	(5)
pJNS12	pBAD-*sfgfp-virB*; Amp^r	(6)
pGB682	pBR322-derived expression vector allowing regulated and dose-dependent recombinant protein expression; *ori* pMB1; Amp^r	(6)
pJH66	pBAD-*linker-sfgfp*; Amp^r	(7)
pJNS18	pBAD-*sfgfp-virB* K152E/R167E; Amp^r	(6)
pJNS43	pBAD-*sfgfp-linker-virB* G91S; Amp^r	This work
pTMG19	pBAD-*sfgfp-linker-virB* R93A; Amp^r	This work
pTMG20	pBAD-*sfgfp-linker-virB* R94A; Amp^r	This work
pTMG21	pBAD-*sfgfp-linker-virB* R95A; Amp^r	This work
pAMO18	pBAD-*sfgfp-linker-virB* T68A; Amp^r	This work
pAMO19	pBAD-*sfgfp-linker-virB* T68S; Amp^r	This work
pJNS22	pACYC177 carrying P*tac-lacZ* with the VirB binding site from P*icsP* (8, 9); pBT-P*icsP* (Cm ^{r})	This work
pHJW20	P*icsP*-*lacZ* reporter plasmid derived from pACYC184; Cm^r	(1)
pMIC18	pHJW20 with VirB boxes 1 and 2 mutated by transition mutations; Cm^r	(8)

Table S2. Primers used in this study

Primer	Sequence 5' to 3'	Description and Use
W43	CTCTACTGTTTCTCCATACCC	Sequencing primer for pBAD-*virB* mutants (I65A, T68A, T68S, & F74A)
W368	TCATTCGCTAGCAAACCACCCCAATATAAGTTTGAG	Sequencing primer for pBAD-*virB* G91S
W453	AGCGAATTCATAAACAGGGTGTGAT	Sequencing primer used for pBAD-*virB* mutants (R93A, R94A, & R95A)
W454	GAAATTCTGGATGGCACTGCTAGAAGAGCATCTGCAA TATATGC	Mutagenic primer used to generate pBAD-*virB* R93A
W455	GAAATTCTGGATGGCACTCGTGCAAGAGCATCTGCAA TATATGC	Mutagenic primer used to generate pBAD-*virB* R94A
W456	GAAATTCTGGATGGCACTCGTAGAGCAGCATCTGCAA TATATGC	Mutagenic primer used to generate pBAD-*virB* R95A
W457	GAGATATTATTTCCTGTGGAACGCTTGC	Primer used to generate megaprimers for pBAD-*virB* mutants (R93A, R94A, & R95A); Sequencing primer for pBAD-*virB* mutants (R93A, R94A, & R95A)
W458	ATTATTTGCACGGCGTCACACTTTGC	Primer for amplification of pBAD-*virB* derivatives (G91S, R93A, R94A, & R95A)
W543	CTAGTCAAAGCTTATGAAGACGATAGATGGCGAGA	Primer for amplification of pBAD-*virB* G91S
W563	CCGAACGAAAAGCGCGACCACATGG	Sequencing primer used for pBAD-*sfgfp-linker-virB* derivatives (T68A & T68S)
W638	TTCTGCGTTCTGATTTAATCTGTATCAGGC	Sequencing primer for pBAD-*virB* mutants (I65A, T68A, T68S, & F74A) and pBAD-*sfgfp-linker-virB* derivatives (T68A & T68S); Primer used to amplify pBAD-*virB* DBM
W651	CGAGACAGATTCTCTTTTTTGGCGATATCCTCATAGG ACATCCC	Mutagenic primer used to generate pBAD-*virB* K152E
W652	GCACTCGTAGAAGAGCATCTGCA	Primer for amplification of pBAD-*virB* K152E and pBAD-*virB* DBM
W653	GGCTGAAAATCTTCTCTCATCCGCC	Primer for amplification of Box 1 mutants and pBAD-*virB* K152E; Sequencing primer for pBAD-*virB* K152E and DBM
W665	GATTAGCGGATCCTACCTGACGC	Sequencing primer for pBAD-*virB* G91S
W721	TGACTAGCTCGAGGTGGATTTGTGCAACGACTTG	Primer used for amplification of pBAD-*sfgfp-linker-virB* derivatives (T68A & T68S)
W722	GTATATCGTTTGCTAGTTTTCTGGC	Primer used for amplification of pBAD-*sfgfp-linker-virB* T68A and T68S
W752	TGCTGCCTGAAAGGCCTCAGTGACTTTCGCGCGAGAC AG	Mutagenic primer used to generate pBAD-*virB* DBM
W841	TACGAACCTGTAATAGGAAGGGAGATTGATGGTAGAATTGA AATTCTGGATAGCACTCGT	Mutagenic primer for amplification of pBAD-*virB* G91S
W900	CATCATCATCATGGTATGGCTAGCG	Primer used to generate megaprimer for pBAD-*virB* mutants (I65A, T68A, T68S, & F74A)
W901	GAATTGTTGTAGCTTTATAGCTTTTATGATATCGGC	Primer for amplification of pBAD-*virB* T68A
W902	CAGAAAATTAAGACCAATACCAAGTTCTCGG	Sequencing primer used for pBAD-*virB* mutants (I65A, T68A, T68S, & F74A)
W921	GAATTGTTGTAGCTTTATAGATTTTATGATATCGGC	Primer for amplification of pBAD-*virB* T68S
W925	GCTTTATAGTTTTTATGGCATCGGCTAGTG	Primer for amplification of pBAD-*virB* I65A
W926	CTATTACAGGGAAGGCTTGTTGTAGC	Primer for amplification of pBAD-*virB* F74A

Table S3. Complete statistics for DRaCALA competition assay

Competition Assay		no protein	VirB + cold competitor				
			no cold	CTP	UTP	ATP	GTP
no protein			<0.001 *	<0.001 *	<0.001 *	<0.001 *	<0.001 *
VirB + cold competitor	no cold			<0.001 *	0.054	0.983	0.063
	CTP				<0.001 *	<0.001 *	<0.001 *
	UTP					1	1
	ATP						1
	GTP						

Significance was determined using a one-way ANOVA with post hoc Bonferroni. Asterisks indicate $p<0.05$. Grey boxes represent data that was not compared.

Table S4. Complete statistics for anti-silencing of P*icsP*

VirB Anti-silencing Activity	WT	Empty	K152E	DBM	G91S	R93A	R94A	R95A	T68A	T68S	I65A	F74A
**WT**		<0.001 *	<0.001 *	<0.001 *	<0.001 *	<0.001 *	<0.001 *	<0.001 *	<0.001 *	1	<0.001 *	<0.001 *
**Empty**			0.593	1	1	1	1	<0.001 *	0.998	<0.001 *	1	1
**K152E**				0.825	0.798	0.470	0.693	<0.001 *	0.989	<0.001 *	0.766	0.778
**DBM**					1	1	1	<0.001 *	1	<0.001 *	1	1
**G91S**						1	1	<0.001 *	1	<0.001 *	1	1
**R93A**							1	<0.001 *	0.989	<0.001 *	1	1
**R94A**								<0.001 *	1	<0.001 *	1	1
**R95A**									<0.001 *	<0.001 *	<0.001 *	<0.001 *
**T68A**										<0.001 *	1	1
**T68S**											<0.001 *	<0.001 *
**I65A**												1
**F74A**												

Significance was determined using a one-way ANOVA with post hoc Tukey HSD. Asterisks indicate $p<0.05$. Grey boxes represent data that was not compared.

Table S5. Complete statistics for Congo red binding assay

Congo Red Binding Activity (0.2% L-ara)	WT	Empty	K152E	DBM	R93A	R94A	R95A	T68A	T68S	I65A	F74A
WT		<0.001 *	<0.001 *	<0.001 *	<0.001 *	<0.001 *	0.002*	<0.001 *	0.759	<0.001 *	<0.001 *
Empty			<0.001 *	1	1	1	<0.001 *	0.993	<0.001 *	1	1
K152E				<0.001 *	<0.001 *	<0.001 *	<0.001 *	<0.001 *	0.010*	<0.001 *	<0.001 *
DBM					1	1	<0.001 *	0.999	<0.001 *	1	1
R93A						1	<0.001 *	0.995	<0.001 *	1	1
R94A							<0.001 *	0.999	<0.001 *	1	1
R95A								<0.001 *	<0.001 *	<0.001 *	<0.001 *
T68A									<0.001 *	1	0.999
T68S										<0.001 *	<0.001 *
I65A											1
F74A											

Congo Red Binding Activity (0.2% Glu)	WT	Empty	K152E	DBM	R93A	R94A	R95A	T68A	T68S	I65A	F74A
WT		0.853	1	1	1	1	1	1	1	0.999	1
Empty			0.991	0.988	0.984	0.967	0.957	0.946	0.947	0.999	0.976
K152E				1	1	1	1	1	1	1	1
DBM					1	1	1	1	1	1	1
R93A						1	1	1	1	1	1
R94A							1	1	1	1	1
R95A								1	1	1	1
T68A									1	1	1
T68S										1	1
I65A											1
F74A											

Significance was determined using a one-way ANOVA with post hoc Tukey HSD. Asterisks indicate p<0.05. Grey boxes represent data that was not compared.

Table S6. Complete statistics for fluorescence microscopy distributions

Focus Formation	WT	Empty	GFP	DBM	R93A	R94A	R95A	T68A	T68S
WT		<.001 *	<.001 *	<.001 *	<.001 *	<.001 *	<.001 *	<.001 *	.054
Empty			<.001 *	<.001 *	<.001 *	<.001 *	<.001 *	<.001 *	<.001 *
GFP				<.001 *	<.001 *	<.001 *	<.001 *	.147	<.001 *
DBM					.965	.190	0.099	<.001 *	<.001 *
R93A						.211	0.537	<.001 *	<.001 *
R94A							0.118	.024*	<.001 *
R95A								<.001 *	<.001 *
T68A									<.001 *
T68S									

Significance was determined using a Kolmogorov-Smirnov test with post hoc Bonferroni. Asterisks indicate p<0.05. Grey boxes represent data that was not compared.

Table S7. Complete statistics for *in vivo* DNA binding assay

DNA Binding Activity	WT	Empty	K152E	DBM	R93A	R94A	R95A	T68A	T68S	I65A	F74A
**WT**		<0.001 *	<0.001 *	<0.001 *	<0.001 *	<0.001 *	0.004*	<0.001 *	0.994	<0.001 *	<0.001 *
**Empty**			0.008*	1	1	0.543	<0.001 *	0.242	<0.001 *	0.203	0.110
**K152E**				0.043*	0.021*	0.606	0.495	0.892	<0.001 *	0.926	0.986
**DBM**					1	0.912	<0.001 *	0.642	<0.001 *	0.578	0.381
**R93A**						0.866	<0.001 *	0.442	<0.001 *	0.384	0.229
**R94A**							0.007*	1	<0.001 *	1	0.997
**R95A**								0.025*	<0.001 *	0.032*	0.065*
**T68A**									<0.001 *	1	1
**T68S**										<0.001 *	<0.001 *
**I65A**											1
**F74A**											

Significance was determined using a one-way ANOVA with post hoc Tukey HSD. Asterisks indicate p<0.05. Grey boxes represent data that was not compared.

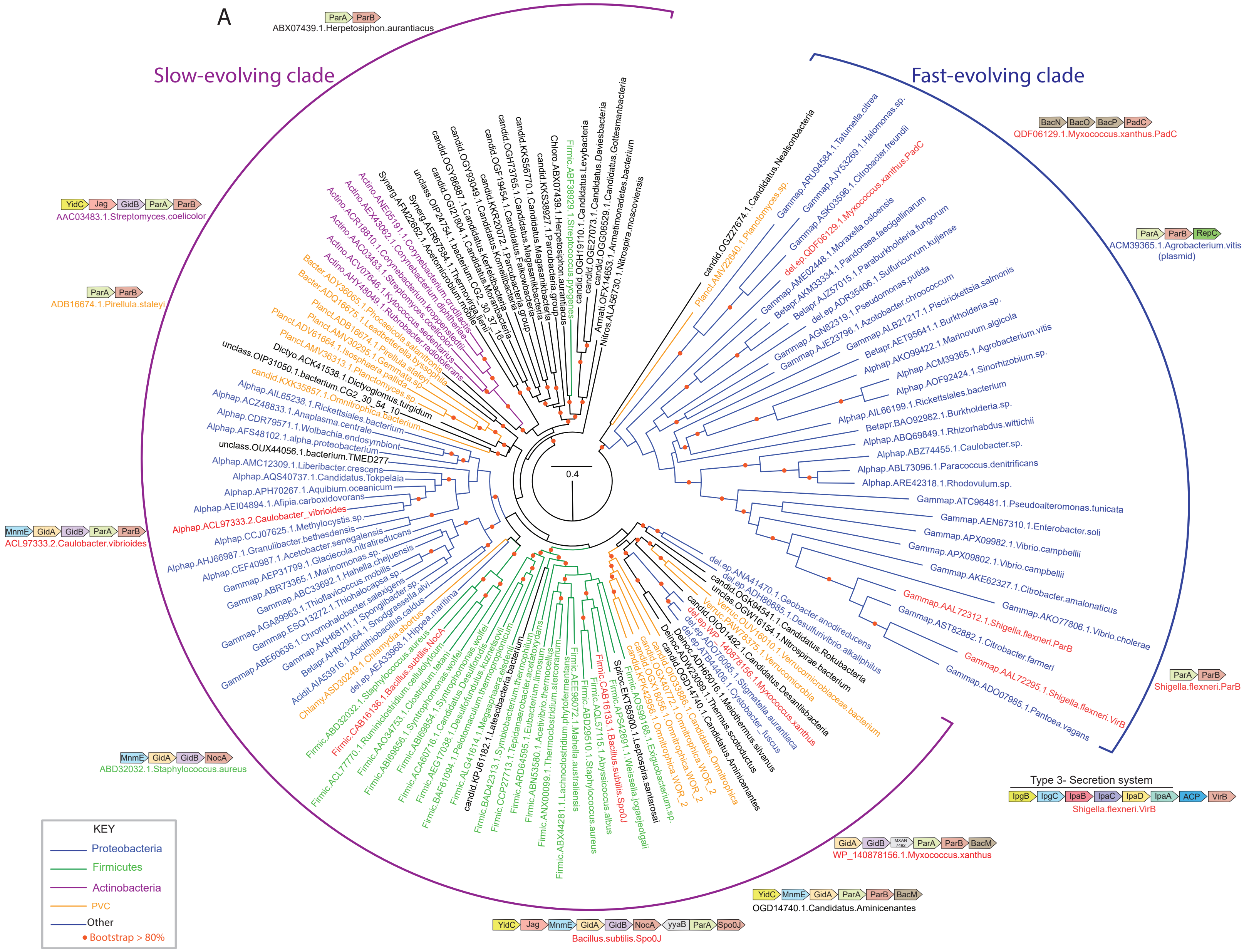

Figure S1. Full maximum likelihood tree of ParB protein sequences including the VirB-like fast-evolving sequences. (A) Maximum likelihood tree of ParB protein sequences including the VirB-like fast-evolving sequences. The tree was computed using IQtree and bootstrapped with 1000 replicates. Nodes and branches corresponding to widely represented phylogenetic clades are colored according to the key. Protein sequences are denoted with their clade, gene and species name. The slow- and fast-evolving clades are marked and representative gene neighborhoods of distinct clades of the tree are shown. Genes in gene neighborhoods are shown as box arrows with the arrowhead pointing to the gene at the 3' end. Clade abbreviations include: Acidit: Acidiothiobacillus, Actino: Actinomycetes, Alphap: Alpha-proteobacteria, Armati: Armatimonas, Bacter: Bacteroidetes, Betapr: Beta-proteobacteria, candid: Dark-matter bacteria, Chlamy: Chlamydiae, Chloro: Chloroflexi, Deinoc: Thermus/Deinococcus, del.ep: Delta/epsilon proteobacteria, Dictyo: Dictyoglomus, Firmic: Firmicutes, Gammap: Gamma-proteobacteria, Nitros: Nitrospirae, Planct: Planctomycetes, Spiroc: Spirochaetes, Synerg: Synergistetes, Verruc: Verrucomicrobiae, unclass: Unclassified bacteria.
