# Supplementary material for: VirB, a key transcriptional regulator of Shigella virulence, requires a CTP ligand for its regulatory activities
Source: mBio. 2023 Sep 20;14(5):e01519-23. doi: 10.1128/mbio.01519-23 (PMC10653881; doi:10.1128/mbio.01519-23)
Supplement: Supplemental material part 2 — Figures S2-S5 and references. [file mbio.01519-23-s0002.pdf]

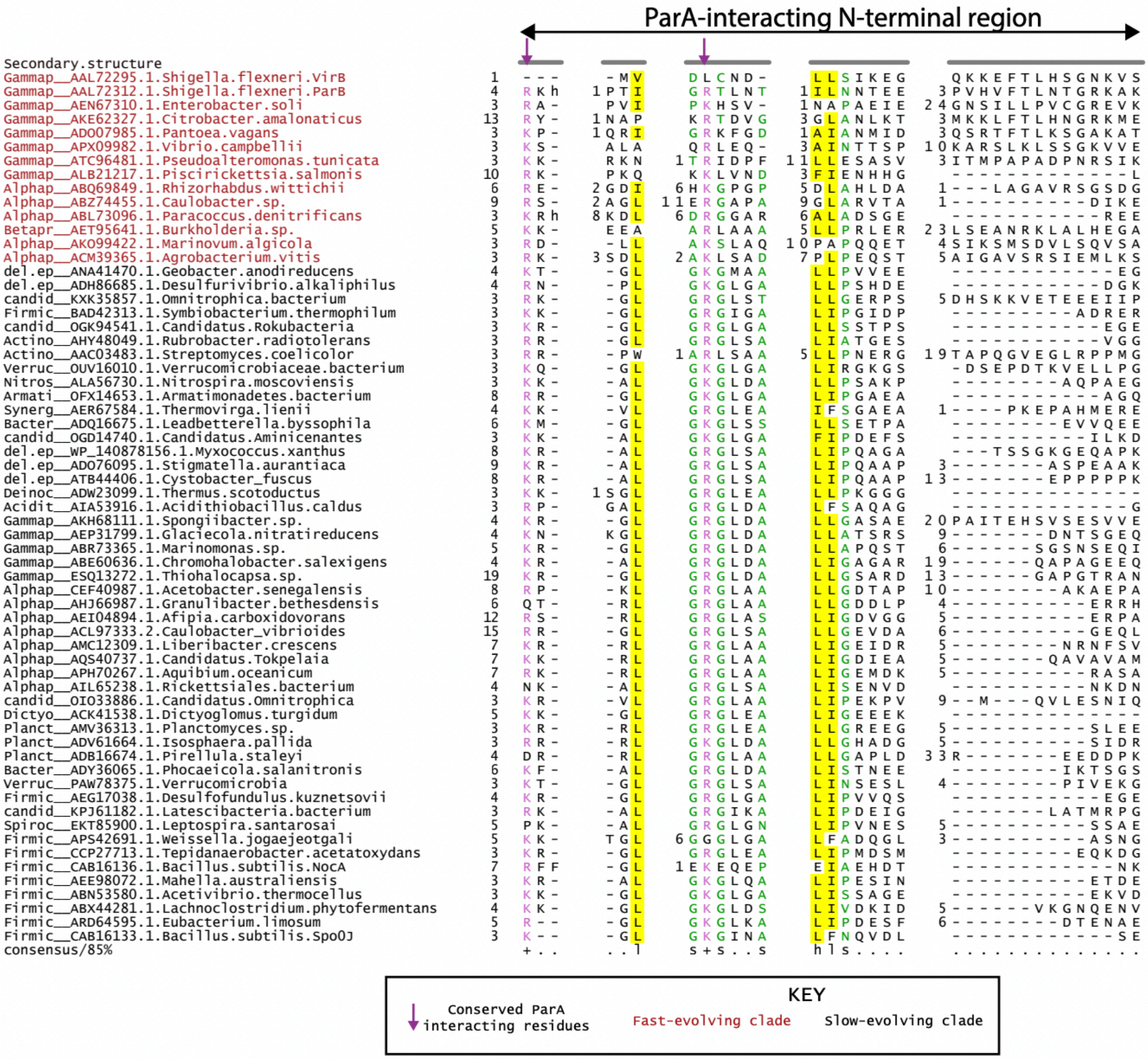

ParA-interacting N-terminal region
Secondary.structure
Gammap__AAL72295.1.Shigella.flexneri.VirB
Gammap__AAL72312.1.Shigella.flexneri.ParB
Gammap__AEN67310.1.Enterobacter.soli
Gammap__AKE62327.1.Citrobacter.amalonaticus
Gammap__ADO07985.1.Pantoea.vagans
Gammap__APX09982.1.Vibrio.campbellii
Gammap__ATC96481.1.Pseudoalteromonas.tunicata
Gammap__ALB21217.1.Piscirickettsia.salmonis
Alphap__ABQ69849.1.Rhizorhabdus.wittichii
Alphap__ABZ74455.1.Caulobacter.sp.
Alphap__ABL73096.1.Paracoccus.denitrificans
Betapr__AET95641.1.Burkholderia.sp.
Alphap__AKO99422.1.Marinovum.algicola
Alphap__ACM39365.1.Agrobacterium.vitis
del.ep__ANA41470.1.Geobacter.anodireducens
del.ep__ADH86685.1.Desulfurivibrio.alkaliphilus
candid__KXK35857.1.Omnitrophica.bacterium
Firmic__BAD42313.1.Symbiobacterium.thermophilum
candid__OGK94541.1.Candidatus.Rokubacteria
Actino__AHY48049.1.Rubrobacter.radiotolerans
Actino__AAC03483.1.Streptomyces.coelicolor
Verruc__OUV16010.1.Verrucomicrobiaceae.bacterium
Nitros__ALA56730.1.Nitrospira.moscoviensis
Armati__OFX14653.1.Armatimonadetes.bacterium
Synerg__AER67584.1.Thermovirga.lienii
Bacter__ADQ16675.1.Leadbetterella.byssophila
candid__OGD14740.1.Candidatus.Aminicenantes
del.ep__WP_140878156.1.Myxococcus.xanthus
del.ep__ADO76095.1.Stigmatella.aurantiaca
del.ep__ATB44406.1.Cystobacter_fuscus
Deinoc__ADW23099.1.Thermus.scotoductus
Acidit__AIA53916.1.Acidithiobacillus.caldus
Gammap__AKH68111.1.Spongiibacter.sp.
Gammap__AEP31799.1.Glaciecola.nitratireducens
Gammap__ABR73365.1.Marinomonas.sp.
Gammap__ABE60636.1.Chromohalobacter.salexigens
Gammap__ESQ13272.1.Thiohalocapsa.sp.
Alphap__CEF40987.1.Acetobacter.senegalensis
Alphap__AHJ66987.1.Granulibacter.bethesdensis
Alphap__AEI04894.1.Afipia.carboxidovorans
Alphap__ACL97333.2.Caulobacter_vibrioides
Alphap__AMC12309.1.Liberibacter.crescens
Alphap__AQS40737.1.Candidatus.Tokpelaia
Alphap__APH70267.1.Aquibium.oceanicum
Alphap__AIL65238.1.Rickettsiales.bacterium
candid__OIO33886.1.Candidatus.Omnitrophica
Dictyo__ACK41538.1.Dictyoglomus.turgidum
Planct__AMV36313.1.Planctomyces.sp.
Planct__ADV61664.1.Isosphaera.pallida
Planct__ADB16674.1.Pirellula.staleyi
Bacter__ADY36065.1.Phocaeicola.salanitronis
Verruc__PAW78375.1.Verrucomicrobia
Firmic__AEG17038.1.Desulfofundulus.kuznetsovii
candid__KPJ61182.1.Latescibacteria.bacterium
Spiroc__EKT85900.1.Leptospira.santarosai
Firmic__APS42691.1.Weissella.jogaejeotgali
Firmic__CCP27713.1.Tepidanaerobacter.acetatoxydans
Firmic__CAB16136.1.Bacillus.subtilis.NocA
Firmic__AEE98072.1.Mahella.australiensis
Firmic__ABN53580.1.Acetivibrio.thermocellus
Firmic__ABX44281.1.Lachnoclostridium.phytofermentans
Firmic__ARD64595.1.Eubacterium.limosum
Firmic__CAB16133.1.Bacillus.subtilis.Spo0J
consensus/85%
KEY
Conserved ParA interacting residues
Fast-evolving clade
Slow-evolving clade

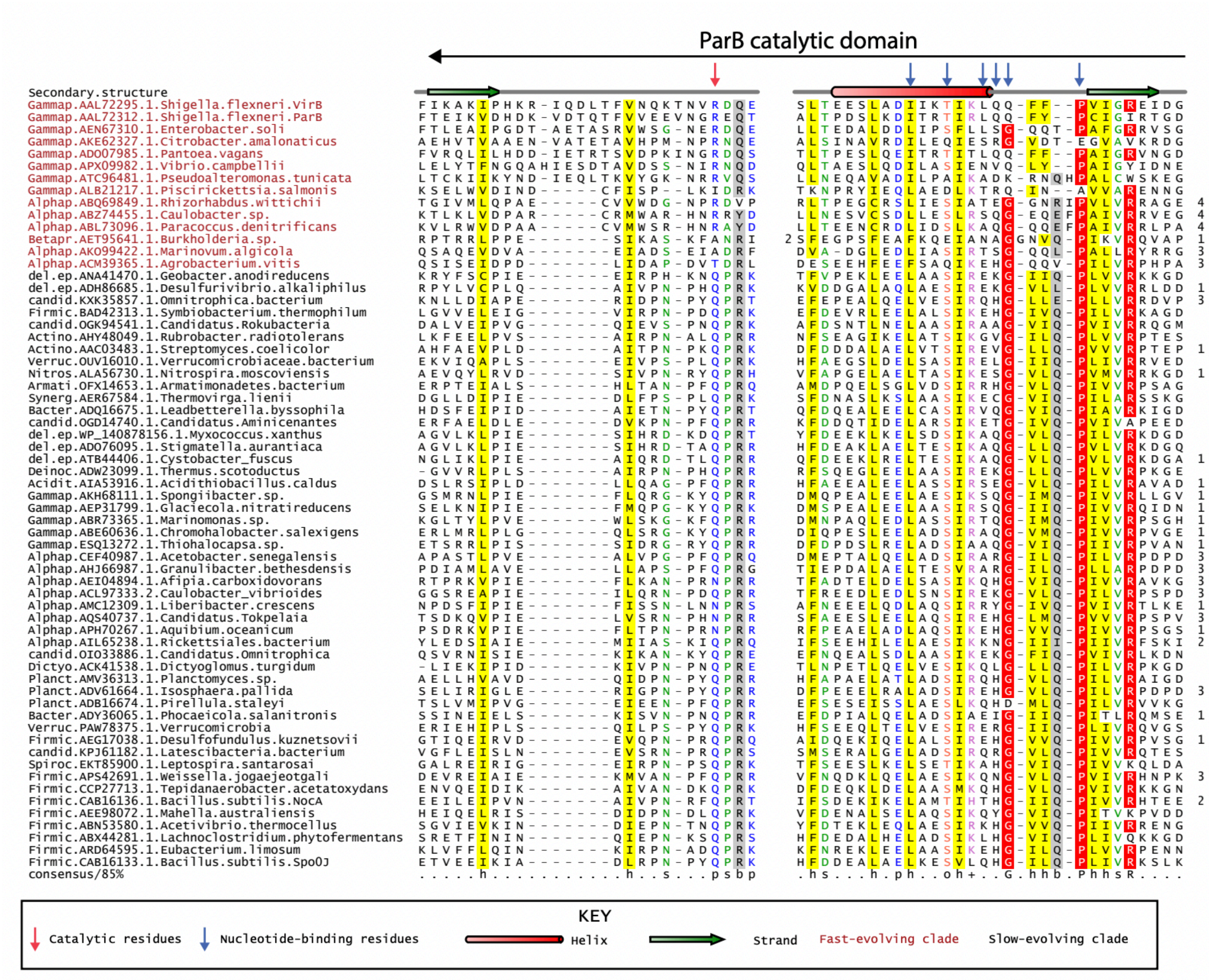
ParB catalytic domain
Secondary.structure
consensus/85%
KEY
Catalytic residues
Nucleotide-binding residues
Helix
Strand
Fast-evolving clade
Slow-evolving clade

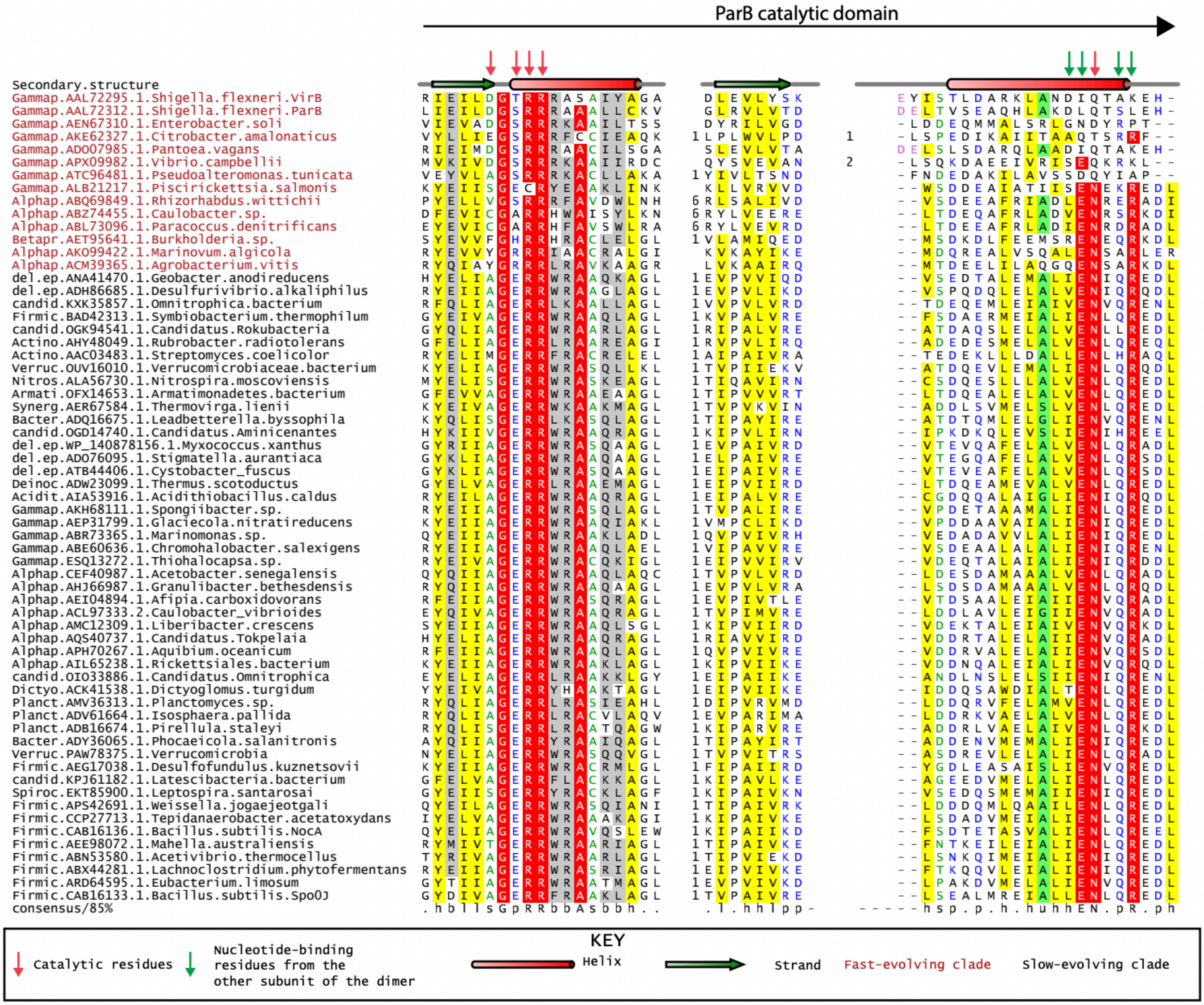
ParB catalytic domain
Secondary.structure
consensus/85%
KEY
Catalytic residues
Nucleotide-binding residues from the other subunit of the dimer
Helix
Strand
Fast-evolving clade
Slow-evolving clade

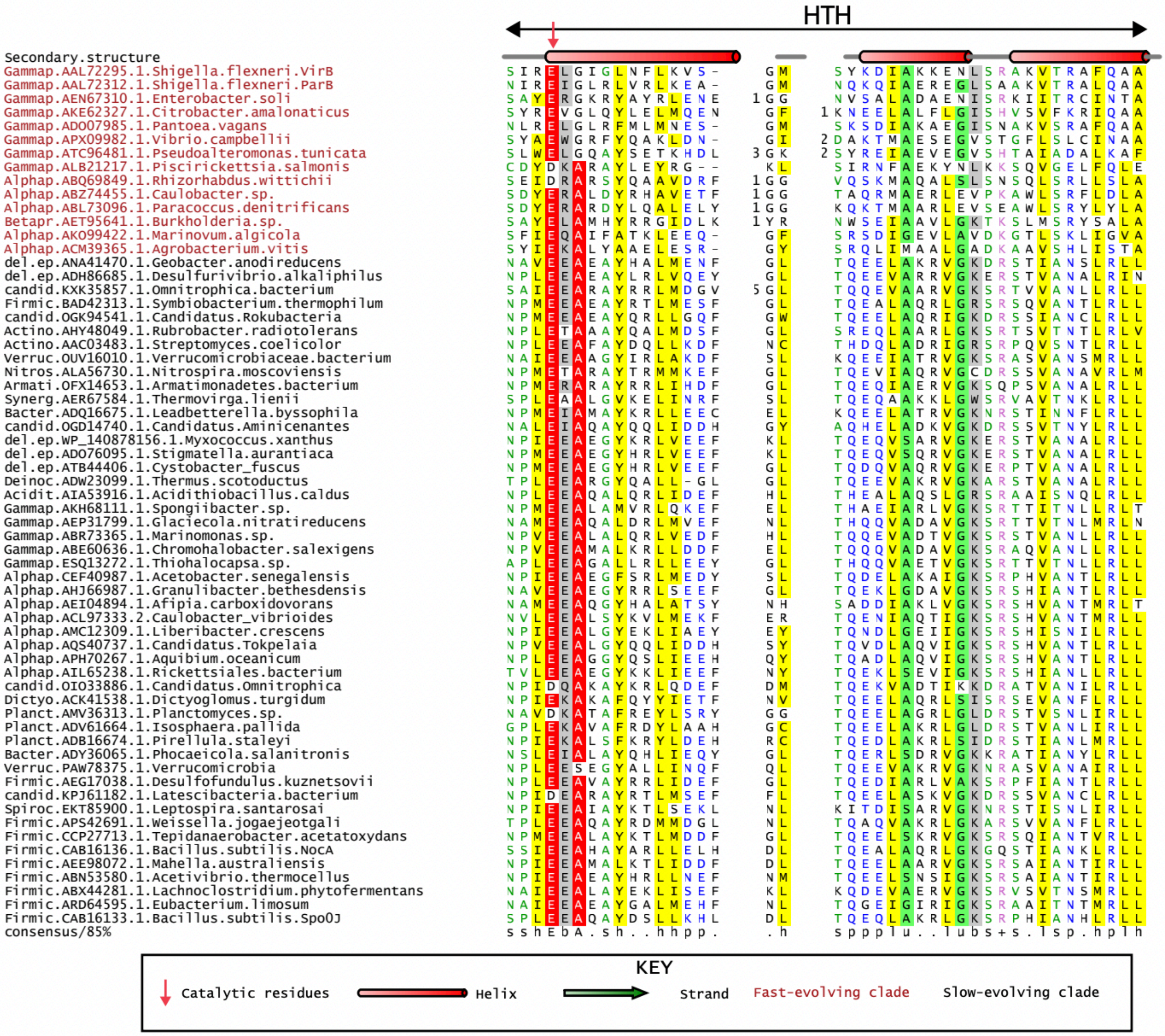
HTH
Secondary.structure
Gammap.AAL72295.1.Shigella.flexneri.VirB
Gammap.AAL72312.1.Shigella.flexneri.ParB
Gammap.AEN67310.1.Enterobacter.soli
Gammap.AKE62327.1.Citrobacter.amalonaticus
Gammap.ADO07985.1.Pantoea.vagans
Gammap.APX09982.1.Vibrio.campbellii
Gammap.ATC96481.1.Pseudoalteromonas.tunicata
Gammap.ALB21217.1.Piscirickettsia.salmonis
Alphap.ABQ69849.1.Rhizorhabdus.wittichii
Alphap.ABZ74455.1.Caulobacter.sp.
Alphap.ABL73096.1.Paracoccus.denitrificans
Betapr.AET95641.1.Burkholderia.sp.
Alphap.AKO99422.1.Marinovum.algicola
Alphap.ACM39365.1.Agrobacterium.vitis
del.ep.ANA41470.1.Geobacter.anodireducens
del.ep.ADH86685.1.Desulfurivibrio.alkaliphilus
candid.KXK35857.1.Omnitrophica.bacterium
Firmic.BAD42313.1.Symbiobacterium.thermophilum
candid.OGK94541.1.Candidatus.Rokubacteria
Actino.AHY48049.1.Rubrobacter.radiotolerans
Actino.AAC03483.1.Streptomyces.coelicolor
Verruc.OUV16010.1.Verrucomicrobiaceae.bacterium
Nitros.ALA56730.1.Nitrospira.moscoviensis
Armati.OFX14653.1.Armatimonadetes.bacterium
Synerg.AER67584.1.Thermovirga.lienii
Bacter.ADQ16675.1.Leadbetterella.byssophila
candid.OGD14740.1.Candidatus.Aminicenantes
del.ep.WP_140878156.1.Myxococcus.xanthus
del.ep.ADO76095.1.Stigmatella.aurantiaca
del.ep.ATB44406.1.Cystobacter_fuscus
Deinoc.ADW23099.1.Thermus.scotoductus
Acidit.AIA53916.1.Acidithiobacillus.caldus
Gammap.AKH68111.1.Spongiibacter.sp.
Gammap.AEP31799.1.Glaciecola.nitratireducens
Gammap.ABR73365.1.Marinomonas.sp.
Gammap.ABE60636.1.Chromohalobacter.salexigens
Gammap.ESQ13272.1.Thiohalocapsa.sp.
Alphap.CEF40987.1.Acetobacter.senegalensis
Alphap.AHJ66987.1.Granulibacter.bethesdensis
Alphap.AEI04894.1.Afipia.carboxidovorans
Alphap.ACL97333.2.Caulobacter_vibrioides
Alphap.AMC12309.1.Liberibacter.crescens
Alphap.AQS40737.1.Candidatus.Tokpelaia
Alphap.APH70267.1.Aquibium.oceanicum
Alphap.AIL65238.1.Rickettsiales.bacterium
candid.OIO33886.1.Candidatus.Omnitrophica
Dictyo.ACK41538.1.Dictyoglomus.turgidum
Planct.AMV36313.1.Planctomyces.sp.
Planct.ADV61664.1.Isosphaera.pallida
Planct.ADB16674.1.Pirellula.staleyi
Bacter.ADY36065.1.Phocaeicola.salanitronis
Verruc.PAW78375.1.Verrucomicrobia
Firmic.AEG17038.1.Desulfofundulus.kuznetsovii
candid.KPJ61182.1.Latescibacteria.bacterium
Spiroc.EKT85900.1.Leptospira.santarosai
Firmic.APS42691.1.Weissella.jogaejeotgali
Firmic.CCP27713.1.Tepidanaerobacter.acetatoxydans
Firmic.CAB16136.1.Bacillus.subtilis.NocA
Firmic.AEE98072.1.Mahella.australiensis
Firmic.ABN53580.1.Acetivibrio.thermocellus
Firmic.ABX44281.1.Lachnoclostridium.phytofermentans
Firmic.ARD64595.1.Eubacterium.limosum
Firmic.CAB16133.1.Bacillus.subtilis.Spo0J
consensus/85%
s s h E b A . s h . . h h p p .
. h
s p p p l u . . l u b s + s . l s p . h p l h
KEY
Catalytic residues
Helix
Strand
Fast-evolving clade
Slow-evolving clade

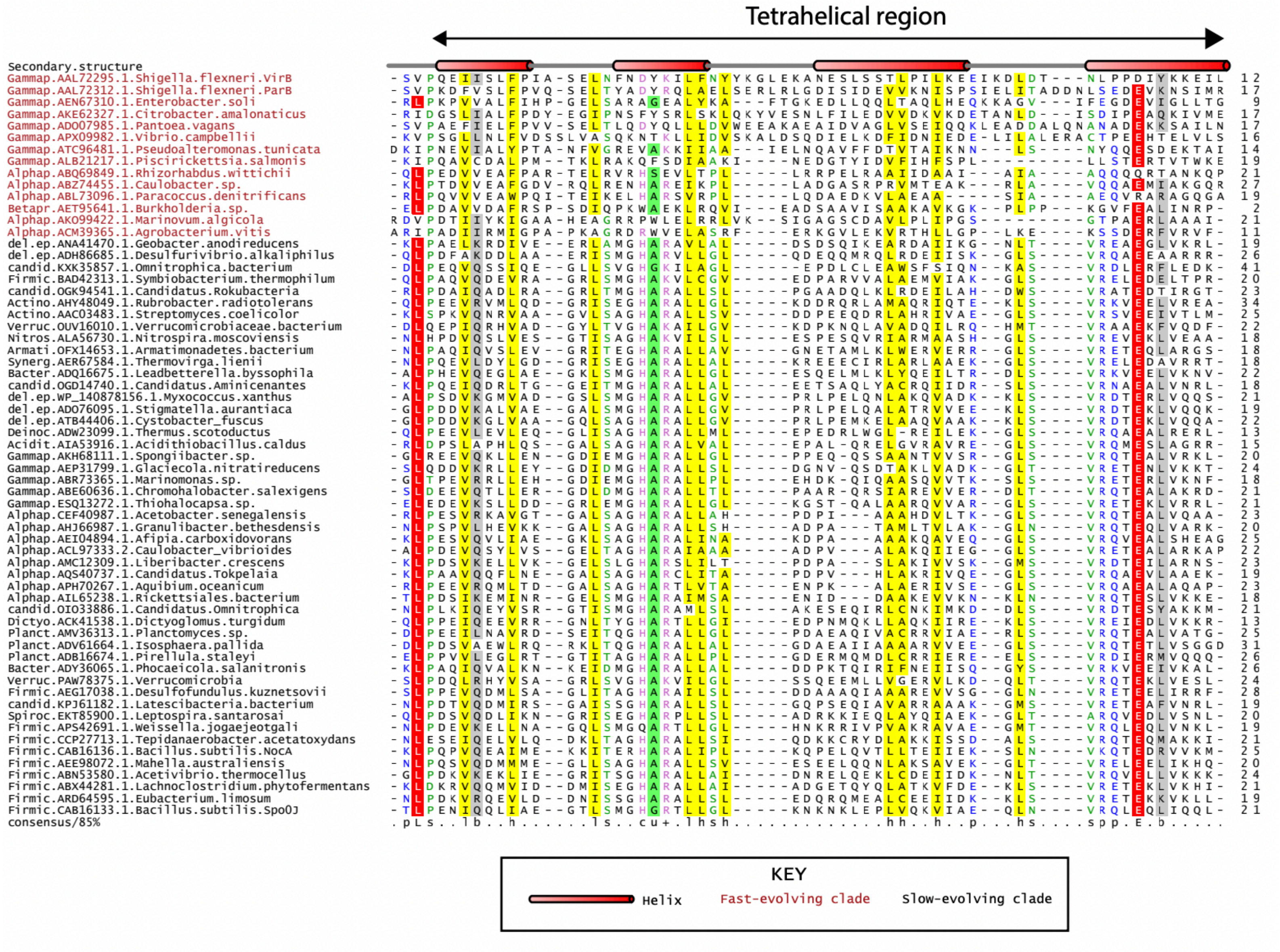
Tetrahelical region
Secondary.structure
consensus/85%
KEY
Helix
Fast-evolving clade
Slow-evolving clade

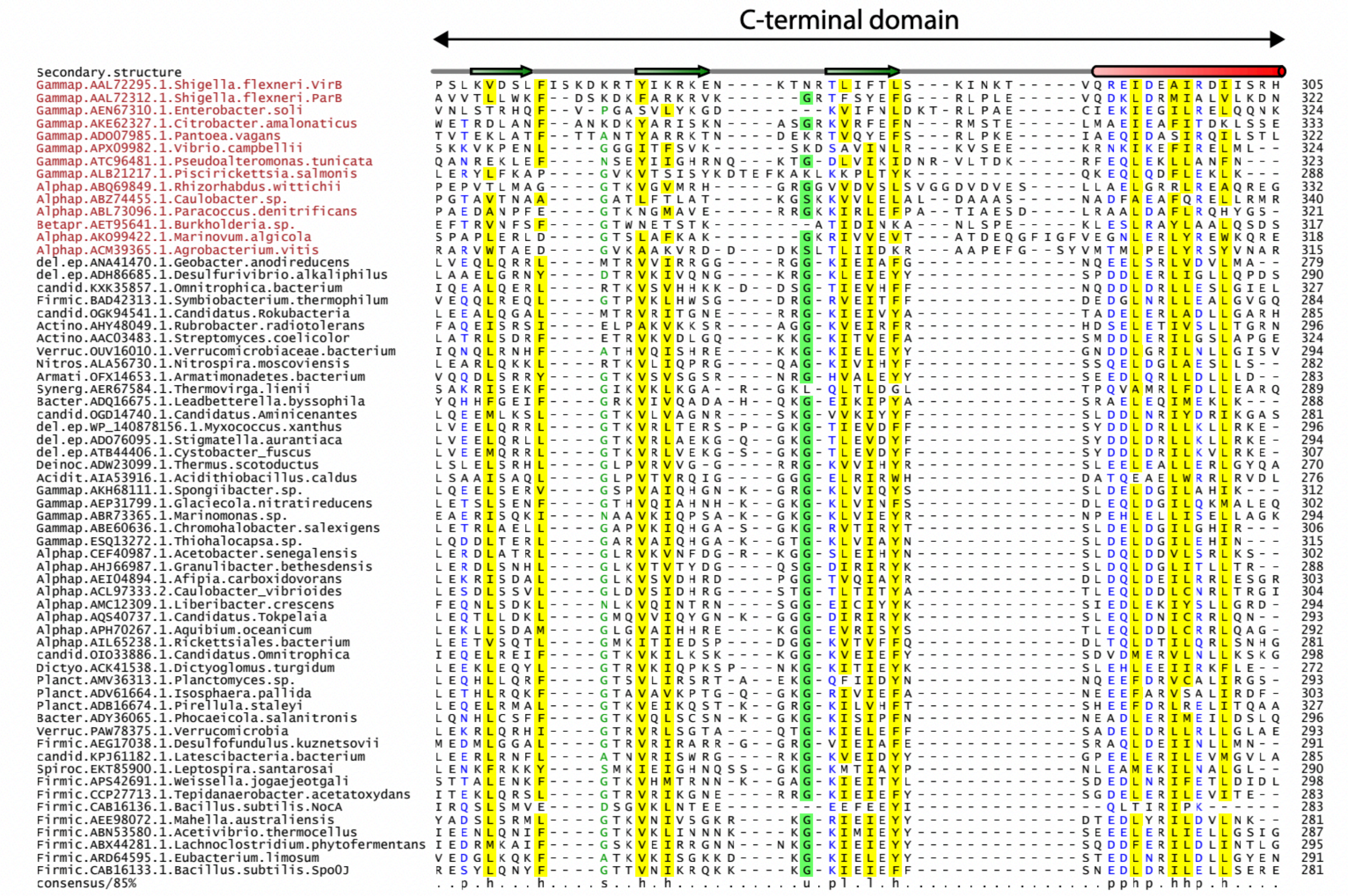

Figure S2. Multiple sequence alignment of slow-evolving (classic) and fast-evolving (e.g., VirB) ParB proteins containing the catalytic ParB, HTH, tetrahelical and C-terminal domains. Taxonomic clade name, Genbank accession, and species name separated by dots are denoted. Secondary structure and the sequence consensus at 85% identity are depicted above and below the alignment, respectively. Coloring of sequence columns is as per the residue consensus abbreviation. Consensus abbreviations and coloring scheme are as follows: h: hydrophobic (FWYILVACM), a: aromatic (FWY) and l: aliphatic (ILV) residues shaded yellow; c: charged (DEHKR) and +: basic (KRH) residues colored magenta, b: big (LIFMWYERKQ) residues shaded grey, o: alcohol-group (ST) residues colored red, p: polar (STECDRKHNQ) residues colored blue, s: small (AGSCDNPTV) residues colored green, and u: tiny (GAS) residues shaded green. Fully conserved residues are shaded red. α-helices and β-strands are depicted as cylinders and arrows, respectively. Numbers between aligned blocks indicate the number of poorly conserved residues that were omitted from the alignment for brevity. Predicted catalytic and nucleotide-interacting positions are indicated. Taxonomic clade abbreviations are as follows; Acidit: Acidithiobacillus, Actino: Actinobacteria, Armati: Armatimonadetes, Bacter: Bacteroidetes, candid: Candidatus (uncultured bacteria), Deinoc: Thermus/Deinococcus, Dictyo: Dictyoglomus, Firmic: Firmicutes, Nitros: Nitrospira, Planct: Planctomycetes, Alphap: Alphaproteobacteria, Betapr: Betaproteobacteria, Gammp: Gamma proteobacteria, del.ep: delta/epsilon protebacteria, Spiroc: Spirochaetes, Synerg: Synergistes, Verruc: Verrucomicrobiae

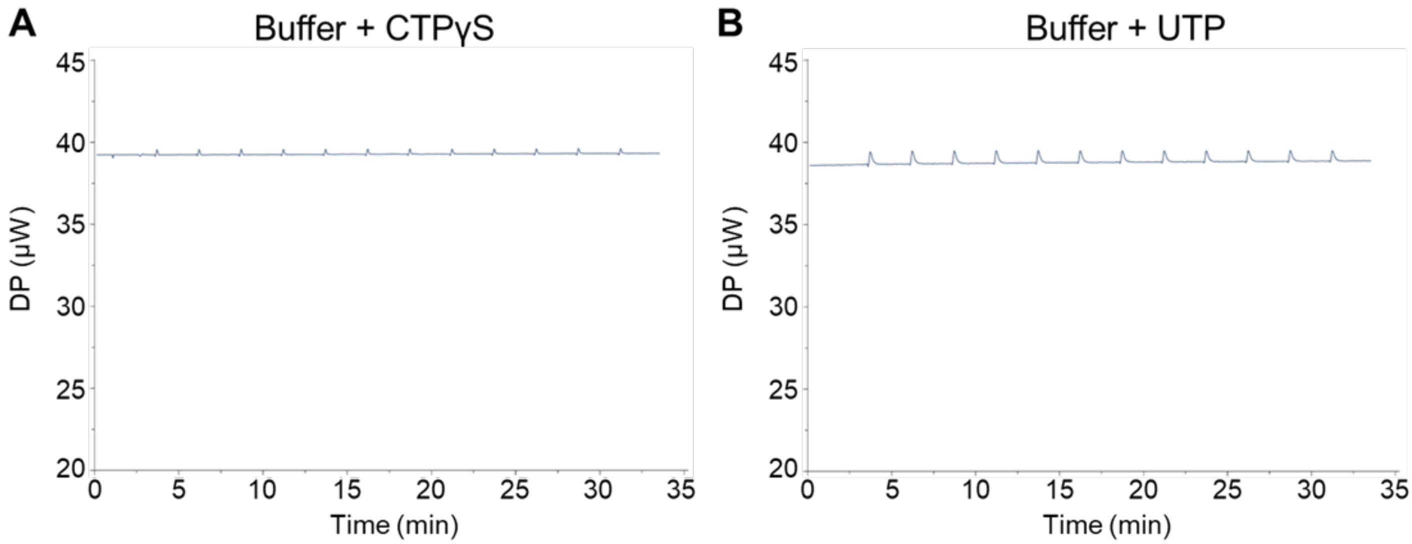

Figure S3. Isothermal Titration Calorimetry buffer-only controls. ITC Measurements of (A) VirB buffer-only and 3 mM CTPγS in the presence of Mg^{2+}, and (B) VirB buffer-only and 3 mM UTP in the presence of Mg^{2+}.

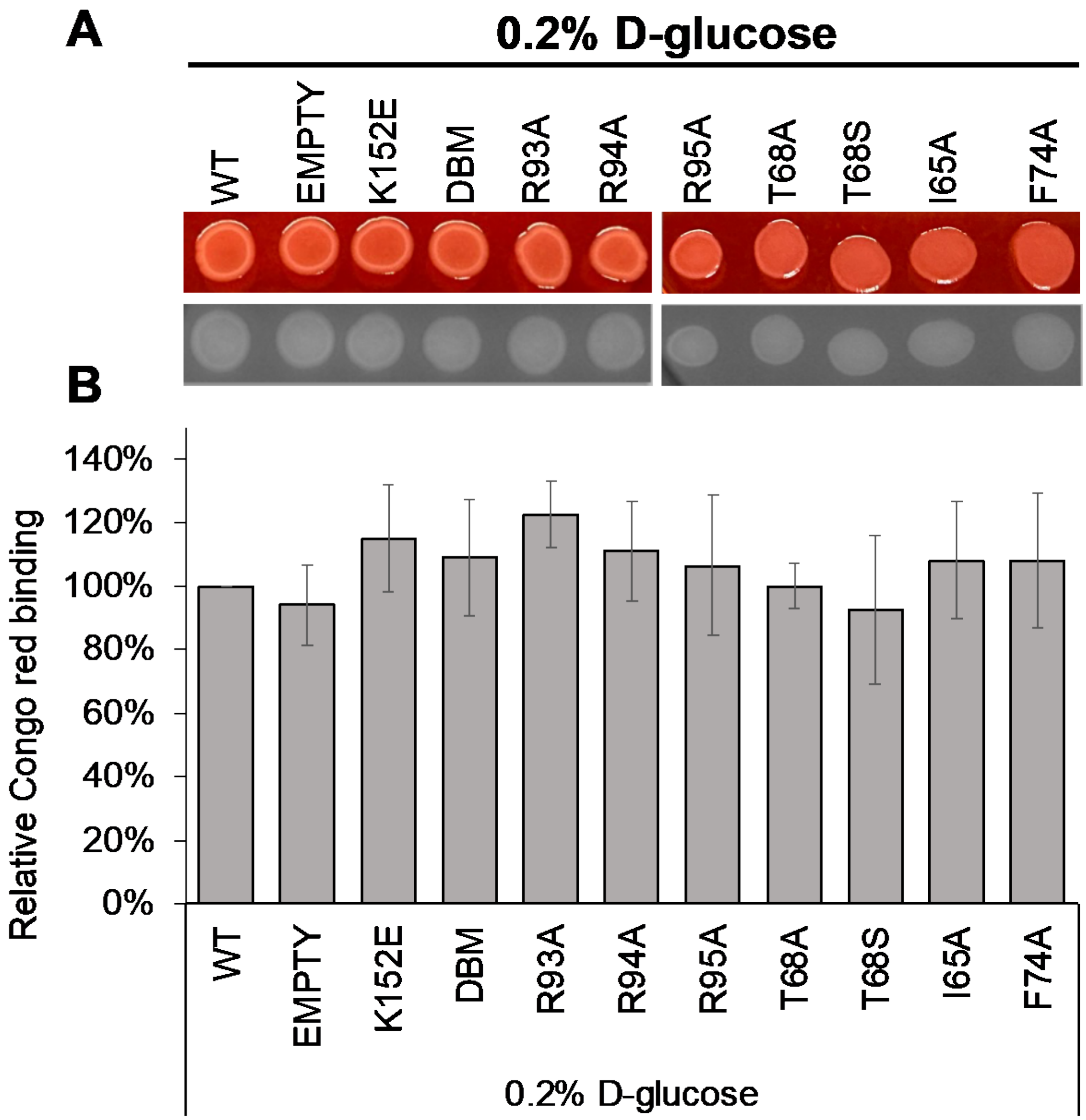

Figure S4. Congo red binding activity of VirB mutants. (A) Congo red binding by *S. flexneri virB*::Tn5 harboring pBAD-VirB derivatives under non-inducing conditions. Images were captured using visible light (top) and blue light (Cy2) (bottom). (B) Quantitative analysis of Congo red binding *S. flexneri virB*::Tn5 harboring pBAD-VirB derivatives (non-induced). Relative Congo red binding was calculated as $[(OD_{498}/OD_{600})/ (\text{average } (OD_{498}/OD_{600})_{2457T\ pBAD})] \times 100$. Assays were completed with three biological replicates and repeated three times. Representative data are shown. Significance was calculated using a one-way ANOVA with post hoc Tukey HSD, $p < 0.05$. *, statistically significant compared to wild-type. Complete statistical analysis is provided in Supplementary Table S5, nothing was deemed significantly different from wild-type.

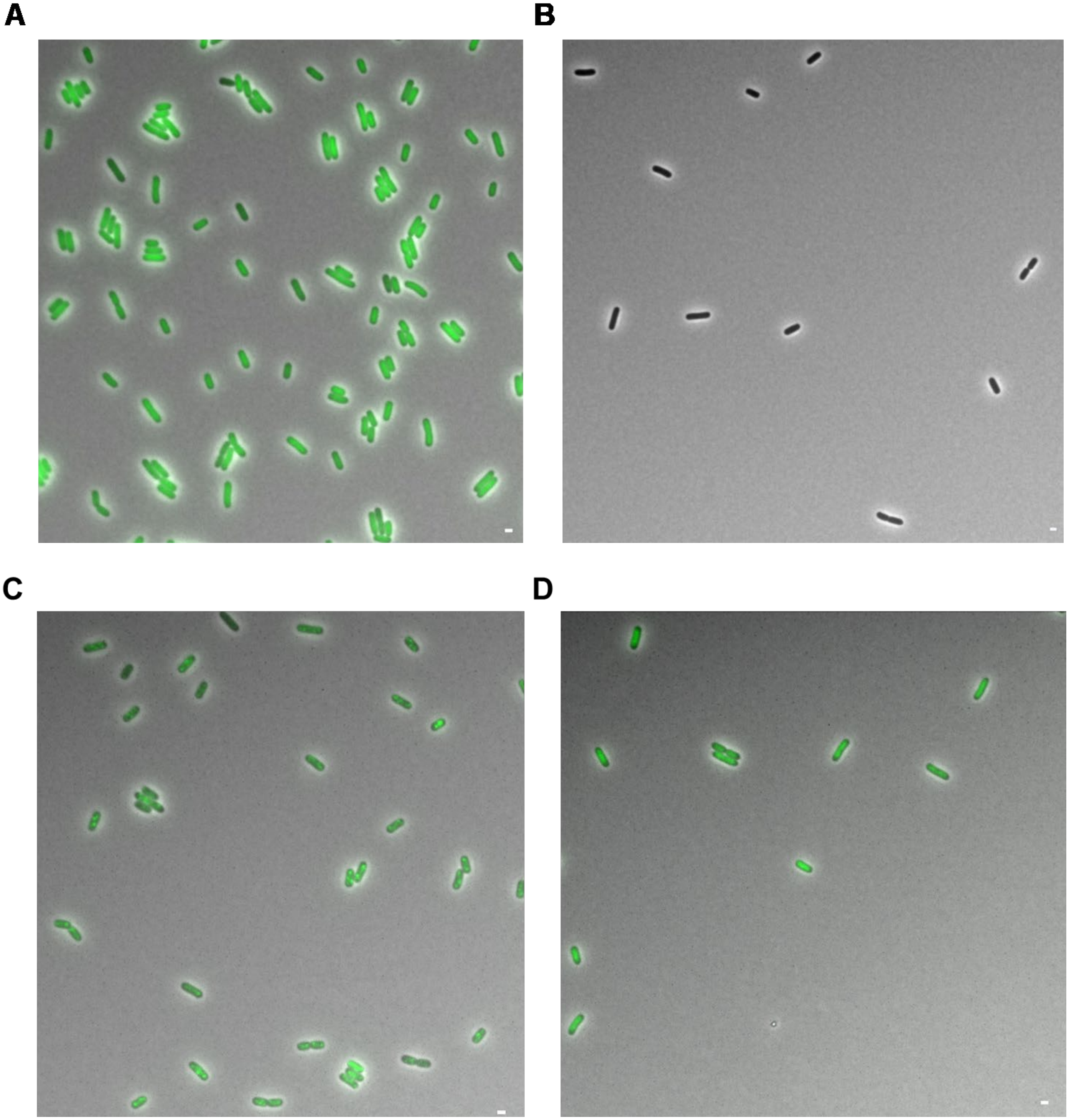
A
B
C
D

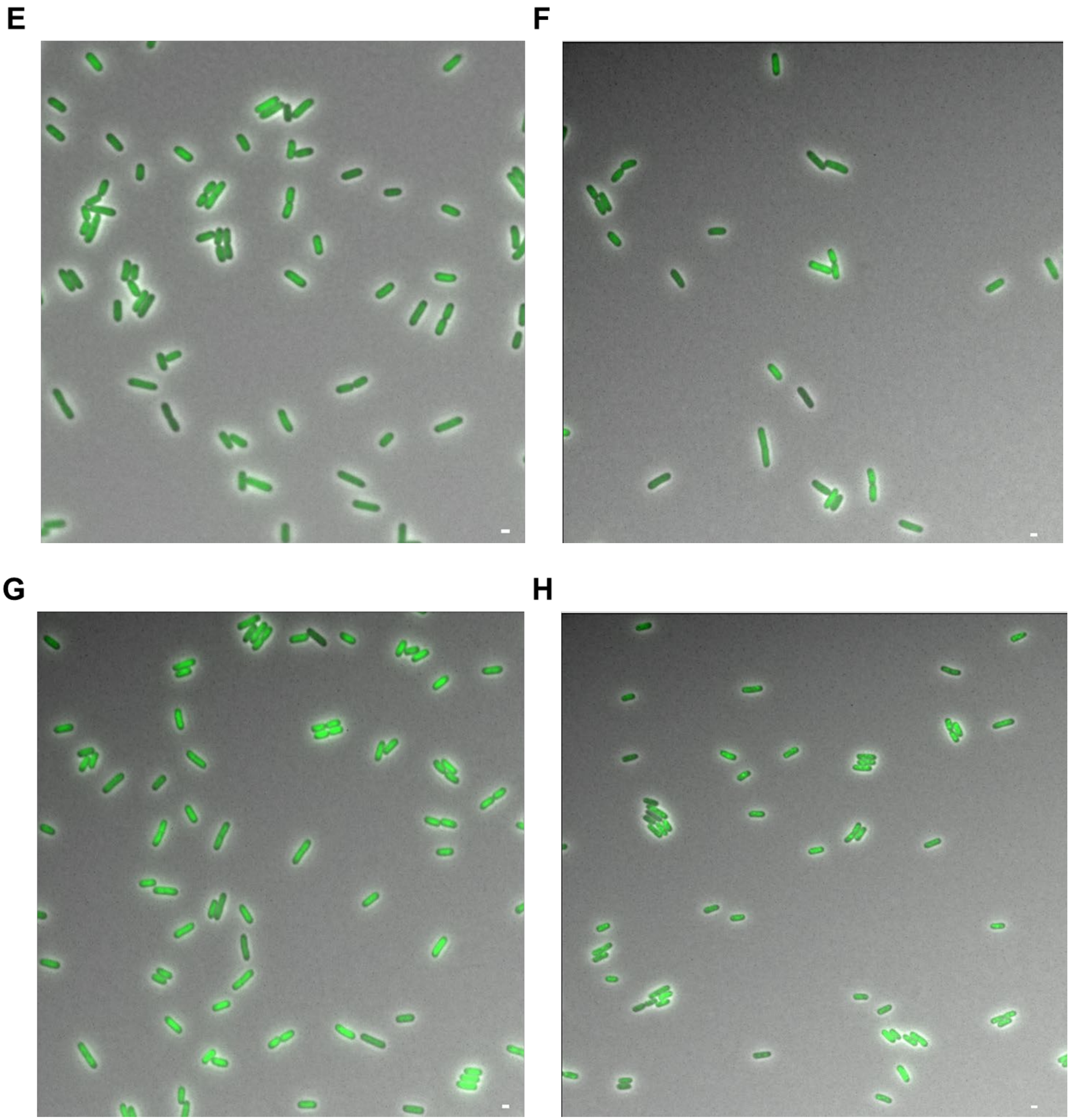
E
F
G
H

I J

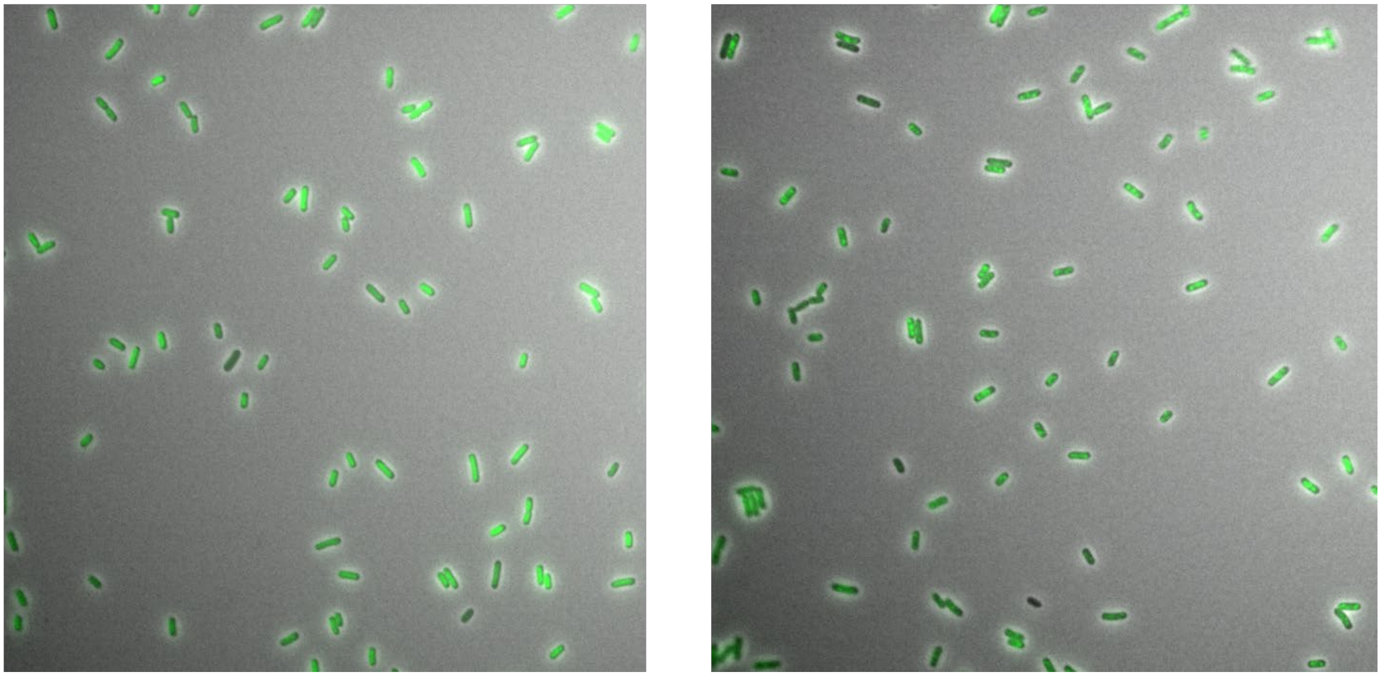

Figure S5. Live cell imaging of GFP-VirB mutants in a *virB* mutant strain of *S. flexneri*. Representative fields of view show either nucleoid-associated or diffuse signals for (A) GFP, (B) EMPTY, (C) GFP-VirB, (D) GFP-VirB DBM, (E) GFP-VirB G91S, (F) GFP-VirB R93A, (G) GFP-VirB R94A, (H) GFP-VirB R95A, (I) GFP-VirB T68A, (J) GFP-VirB T68S. Scale bar represents 1 μm in all images.

References

1. Wing HJ, Yan AW, Goldman SR, Goldberg MB. 2004. Regulation of IcsP, the outer membrane protease of the *Shigella* actin tail assembly protein IcsA, by virulence plasmid regulators VirF and VirB. J Bacteriol 186:699-705.
2. Schuch R, Maurelli AT. 1997. Virulence plasmid instability in Shigella flexneri 2a is induced by virulence gene expression. Infect Immun 65:3686-92.
3. Guzman LM, Belin D, Carson MJ, Beckwith J. 1995. Tight regulation, modulation, and high-level expression by vectors containing the arabinose PBAD promoter. J Bacteriol 177:4121-30.
4. Picker MA, Karney MMA, Gerson TM, Karabachev AD, Duhart JC, McKenna JA, Wing HJ. 2023. Localized modulation of DNA supercoiling, triggered by the *Shigella* anti-silencer VirB, is sufficient to relieve H-NS-mediated silencing. Nucleic Acids Res doi:10.1093/nar/gkad088.
5. Basta DW, Pew KL, Immak JA, Park HS, Picker MA, Wigley AF, Hensley CT, Pearson JS, Hartland EL, Wing HJ. 2013. Characterization of the *ospZ* promoter in *Shigella flexneri* and its regulation by VirB and H-NS. J Bacteriol 195:2562-72.
6. Socea JN, Bowman GR, Wing HJ. 2021. VirB, a key transcriptional regulator of virulence plasmid genes in *Shigella flexneri*, forms DNA-binding site-dependent foci in the bacterial cytoplasm. J Bacteriol 203.
7. Holmes JA, Follett SE, Wang H, Meadows CP, Varga K, Bowman GR. 2016. Caulobacter PopZ forms an intrinsically disordered hub in organizing bacterial cell poles. Proc Natl Acad Sci U S A 113:12490-12495.
8. Castellanos MI, Harrison DJ, Smith JM, Labahn SK, Levy KM, Wing HJ. 2009. VirB alleviates H-NS repression of the *icsP* promoter in *Shigella flexneri* from sites more than one kilobase upstream of the transcription start site. J Bacteriol 191:4047-50.
9. Karney MM, McKenna JA, Weatherspoon-Griffin N, Karabachev AD, Millar ME, Potocek EA, Wing HJ. 2019. Investigating the DNA-binding site for VirB, a key transcriptional regulator of *Shigella* virulence genes, using an *in vivo* binding tool. Genes (Basel) 10.
